# Loss of Drosophila Clueless differentially affects the mitochondrial proteome compared to loss of Sod2 and Pink1

**DOI:** 10.3389/fphys.2022.1004099

**Published:** 2022-10-26

**Authors:** Aditya Sen, Rachel T. Cox

**Affiliations:** ^1^ Department of Biochemistry and Molecular Biology, Uniformed Services University, Bethesda, MD, United States; ^2^ Henry M. Jackson Foundation, Bethesda, MD, United States

**Keywords:** mitochondria, Clueless, SOD2, PINK1, mitochondrial proteome, drosophila, respiratory chain complexes

## Abstract

Mitochondria contain their own DNA, mitochondrial DNA, which encodes thirteen proteins. However, mitochondria require thousands of proteins encoded in the nucleus to carry out their many functions. Identifying the definitive mitochondrial proteome has been challenging as methods isolating mitochondrial proteins differ and different tissues and organisms may have specialized proteomes. Mitochondrial diseases arising from single gene mutations in nucleus encoded genes could affect the mitochondrial proteome, but deciphering which effects are due to loss of specific pathways or to accumulated general mitochondrial damage is difficult. To identify specific *versus* general effects, we have taken advantage of mutations in three Drosophila genes, *clueless*, *Sod2,* and *Pink1*, which are required for mitochondrial function through different pathways. We measured changes in each mutant’s mitochondrial proteome using quantitative tandem mass tag mass spectrometry. Our analysis identified protein classes that are unique to each mutant and those shared between them, suggesting that some changes in the mitochondrial proteome are due to general mitochondrial damage whereas others are gene specific. For example, *clueless* mutants had the greatest number of less and more abundant mitochondrial proteins whereas loss of all three genes increased stress and metabolism proteins. This study is the first to directly compare *in vivo* steady state levels of mitochondrial proteins by examining loss of three pathways critical for mitochondrial function. These data could be useful to understand disease etiology, and how mutations in genes critical for mitochondrial function cause specific mitochondrial proteomic changes as opposed to changes due to generalized mitochondrial damage.

## 1 Introduction

Mitochondria are highly dynamic and multifunctional organelles, responsible for producing the majority of cellular ATP through oxidative phosphorylation. Due to their symbiotic origin of evolution, mitochondria contain their own genome and cannot persist without sufficient nuclear contribution (Margulis 1970; Andersson et al., 1998). Mitochondrial DNA (mtDNA) encodes 13 polypeptides and 2 rRNAs and 22 tRNAs required for the translation of the 13 polypeptides. Mitochondria are involved in many processes, such as metabolism, mitochondrial protein import, fission/fusion, and steroid biosynthesis. To carry out these pathways, mitochondria require over one thousand nucleus-encoded proteins for normal function (Area-Gomez and Schon 2014). Recent studies analyzing the mitochondrial proteome in vertebrates and invertebrates have revealed that the number of so called ‘mitochondrial proteins’ is variable with the composition and numbers varying depending on the source of material and the isolation methods. In fact, it is challenging to define the dynamic nature of the mitochondrial proteome in normal and diseased tissue using different computational algorithms and molecular techniques (Calvo and Mootha 2010).

Mitochondrial diseases often arise from single gene mutations affecting specific processes, but these mutations could also affect protein levels, protein import, or protein turnover in unexpected ways. Symptoms and severity of mitochondrial diseases are complex and sometimes hard to diagnose (Grier et al., 2018; Forny et al., 2021). For researchers modeling mitochondrial disease, parsing the direct effects of a mutation *versus* indirect global effects due to general mitochondrial dysfunction can be challenging. The relatively simple genetics of Drosophila, combined with its usefulness as a model for mitochondrial disease, presents an opportunity to compare how single gene mutations affect mitochondrial proteomes (Sen and Cox 2017).

We have taken advantage of mutations in three well-described genes critical for mitochondrial function, *clueless* (*clu*), *Superoxide dismutase 2* (*Sod2*) and *PTEN-induced putative kinase 1* (*Pink1*), to analyze how loss of each pathway affects the mitochondrial proteome. Clueless (Clu) is a nucleus-encoded ribonucleoprotein that forms mitochondrion-associated particles (Cox and Spradling 2009; Sheard et al., 2020). *clu* mutant flies are sick, sterile, short lived and have Parkinsonism-like phenotypes (Cox and Spradling 2009; Sen et al., 2013). CluH-Knockout (*Cluh-KO*) mutant mice die shortly after birth (Schatton et al., 2017). In addition, *clu* mutant flies and *Cluh-KO* mice have reduced mitochondrial protein (Sen et al., 2015; Schatton et al., 2017). Sod2 scavenges mitochondrial free radicals (Weisiger and Fridovich 1973). *Sod2* null mutant flies only live 24 h and have increased oxidative damage and decreased ATP (Duttaroy et al., 2003; Paul et al., 2007; Godenschwege et al., 2009). Pink1 is a component of the mitophagy pathway used to cull damaged mitochondria (Pickles et al., 2018). Upon damage, Pink1 is stable in the mitochondrial outer membrane and recruits Parkin, an E3 ubiquitin ligase, ultimately leading to degradation of the organelle through mitophagy (Narendra et al., 2010; Ziviani et al., 2010). Pink1 mutant flies have shortened lifespans and mitochondrial damage (Clark et al., 2006; Park et al., 2006; Yang et al., 2006). Disease-causing mutations in CLUH have not yet been identified, possibly because loss of CluH in mouse is nonviable (Schatton et al., 2017). Mutations in the human orthologs SOD2 and PINK1 are known to cause mitochondrial damage and disease in people (Nomiyama et al., 2003; Valente et al., 2004; Mollsten et al., 2007).

In our current study we examined mitochondrial-associated protein abundance in *clu*, *Sod2*, and *Pink1* null mutant flies using tandem mass tag (TMT) quantitative mass spectrometry analysis. Our TMT-mass spectrometry analysis showed that specific subsets of mitochondrial proteins are either less or more abundant depending on the mutation. We analyzed protein differences using Search Tool for the Retrieval of Interacting Genes/Proteins (STRING), Gene ontology (GO) and Protein Annotation through Evolutionary Relationship (PANTHER) analyses (Chen et al., 2013; Szklarczyk et al., 2015; Kuleshov et al., 2016). This revealed that loss of Sod2 had little effect on protein reduction, whereas loss of Clu and Pink1 decreased proteins involved in mitochondrial translation. However, Clu loss specifically decreased protein abundance of mitochondrial respiratory complex proteins. For more abundant proteins, Clu, Sod2, and Pink1 loss increased proteins involved in stress response, such as metabolism and protein folding. However, Clu loss also specifically increased the abundance of proteins involved in vesicle transport and cytoskeletal organization. In addition, using qPCR we found that several Clu low abundance candidates had increased transcript levels in all three genotypes. Finally, since Clu is a ribonucleoprotein, we used Clu immunoprecipitation and RT-PCR to identify five less abundant mitochondrial respiratory complex proteins that bind Clu, which could explain why these proteins have low abundance with Clu loss. Thus, using TMT mass spectrometry, we demonstrated that there appears to be non-specific effects on the mitochondrial proteome due to general mitochondrial dysfunction, and Clu-specific effects on mitochondrial respiratory proteins (less abundant) and proteins involved in vesicle transport and cytoskeletal organization (more abundant). These results help elucidate how single gene mutations responsible for mitochondrial disease cause overlapping and specific symptoms.

## 2 Materials and methods

### 2.1 Fly stocks

The following fly stocks were used for experiments: *w* ^*1118*^ (Wildtype), *clueless* ^*d08713*^ */Cyo Act GFP* (Cox and Spradling 2009)*. Sod2* ^*Δ2*^/*Cyo Act GFP and Pink1* ^*B9*^/*FM7i* were obtained from the Bloomington Drosophila Stock Center. Flies were reared on standard cornmeal fly media at 22 or 25°C.

### 2.2 Preparation of mitochondrial extract for iTRAQ

50 mg of frozen adult flies were homogenized, using a 1 ml Wheaton glass homogenizer and a loose pestle (VWR, Randor, PA), in 1 ml of complete Mitochondrial Isolation Buffer [MIB: 250 mM Sucrose, 10 mM tris-Cl (pH 7.4), 5 mM EDTA, 15 mM MgCl_2_, 1X protease inhibitor cocktail (PIC) and 1 mM DTT]. Nuclei and unbroken cells were removed by centrifuging twice at 1,700 g for 10 min. Crude supernatant was spun again at 10,000 g for 15 min. Supernatant (cytosolic fraction) was removed carefully without disturbing the pellet (mitochondria). The mitochondrial pellet was washed twice with 1 ml MIB without PIC and DTT. The pellet was immediately frozen in dry ice and stored at −80°C until further processing. For mitochondrial protein extraction, frozen mitochondria were suspended in RIPA buffer (Millipore Sigma Cat #R0278) containing 1% sodium dodecyl sulfate (SDS) and kept on ice for 5 min followed by boiling the sample for 5 min. Extract was then spun at 16,000 g for 10 min and supernatant was removed to fresh tubes. Protein concentrations were measured using BCA reagent (Pierce BCA protein assay Kit, Thermo Fisher Scientific, Waltham, MA, United States), aliquoted and flash frozen on dry ice and stored at −80°C for further use. 50 µg of protein samples were sent for quantitative (TMT) mass spectrometry analysis.

### 2.3 Processing of protein samples for TMT labeling and fractionation

Protein samples were reduced and alkylated using DTT [15 mg/ml in 100 mM Triethylammonium bicarbonate (TEAB)] and Iodoacetamide (36 mg/ml in 100 mM TEAB) respectively. Samples were then precipitated with TCA/acetone. Protein pellets were re-constituted in 20% acetonitrile (ACN)/120 mM TEAB and digested with Trypsin/LysC (Promega). Peptides in each sample, 50 µg in 100 µL 110 mM TEAB, were labeled with a TMT 10plex label, in 41 µL acetonitrile, according to the Thermo Fisher protocol. Labeled peptides were combined to a total of 600 µg and split into three aliquots. Peptides were then cleaned (from detergent, small molecules, lipids, and TMT labels access) using Pierce detergent removal columns (Thermo Fisher Scientific, Waltham, MA, United States) and were fractionated by basic reverse phase (bRP) into 24 fractions each containing an average 8,33 µg of protein/fraction.

### 2.4 LCMS/MS and data analysis

Peptides were analyzed by liquid chromatography/tandem mass spectrometry (LCMS/MS) using a nano-LC-Orbitrap-Lumos2 in FTFT interfaced with a nano-LC 1200 system (Thermo Fisher Scientific, Waltham, MA, United States) using reverse-phase chromatography (2%–90% acetonitrile/0.1% FA gradient over 90 min at 300 nL/min) on 75 mm × 150 mm ProntoSIL-120-5-C18 H column (3 µm, 120 Å (BISCHOFF) (Bischoff MZ-ANALYSENTECHNIK GmbH D-55129 Mainz/Germany). Eluted peptides were sprayed directly into a Lumos mass spectrometer through a 1 µm emitter tip (New Objective, Inc. New Littleton, MA) at 2.4 kV. Survey scans (Full MS) were acquired within 350–1,600 Da m/z on an Orbi-trap using the Data dependent Top 15 method with dynamic exclusion of 15 s. Precursor ions were individually isolated with 0.6 Da, then fragmented (MS/MS) using HCD activation collision energy 38. Precursor and fragment ions were analyzed at a resolution of 120,000 AGC, target 1xe6, max IT 50 ms and 50,000, AGC target 1xe5, max IT110 ms, respectively, for three cycles.

Tandem MS2 spectra were processed by Proteome Discoverer (v2.2 Thermo Fisher Scientific). MS/MS spectra were analyzed with Mascot v.2.6.2 (Matrix Science, London, United Kingdom) against 2017RefSeq_83_Drosophila melanogaster and a small DB containing enzymes, BSA, trypsin missed cleavage 2, files RC (recalibration with the same database), precursor mass tolerance 3 ppm, fragment mass tolerance 0.01 Da and Carbamidomethyl on C, TMT 6plex on N-terminus as fixed, and methionine oxidation, Deamidation NQ TMT 6plex on K as variable modifications. Peptides identified in Mascot searches were re-scored with Percolator within the Proteome Discoverer to select identified peptides with a confidence threshold 0.01% False Discovery Rate b (Mascot search with Percolator re-scoring) and to calculate the protein and peptide ratios. Only Peptide Rank 1 was considered. The database search identified ∼6150 proteins at high, medium, and low confidence with at least one peptide identified at 1% FDR Rank1. Ratios were calculated for the average of the two replicates. ANOVA analysis of the individual protein was used as a statistical method to calculate *p* values. Data from each mutant were compared with wild type control. For a detailed analysis of each genotype, raw data were curated into separate excel files along with the abundance ratios and *p*-values. The abundance ratio and *p*-value were converted into their respective logarithmic scales. Next, we used GraphPad Prism to plot the values in a volcano plot with a statistically significant threshold (*p* ≤ 0.05 or −log10[*p*-value] ≥ 1.3] as well as Fold Change cut-off lines on the graphs.

### 2.5 Western blotting

Western blotting was performed as previously described (Sen et al., 2013; Sen et al., 2015). In short, protein samples from adult flies were extracted in a 1X SDS sample buffer. samples were run on 4%–15% TGX gels (Cat # 4561086, Bio-rad Laboratories, Hercules, CA) and transferred on to a nitrocellulose membrane (Thermo Fisher Scientific, Waltham, MA, United States) using a Trans-blot Semi-dry apparatus (Cat # 1073940, Bio-rad Laboratories, Hercules, CA). After blocking, blots were probed against appropriate primary and secondary antibodies. anti-Clu (1:15,000) (Cox and Spradling 2009), anti-TOM20 (1:2000, Santa Cruz Biotechnology, Dallas, TX), anti-ATP5A (1:100,000, Abcam, Cambridge, United Kingdom). The following antibodies were kindly provided by Dr. Edward Owusu-Ansah from Columbia University Medical Center, NY: anti-ND-ASHI (1:3,000), anti-ND-SGDH (1:3,000), anti-ND-17 (1:3,000), anti-ND-17.2 (1:3,000) and anti-UQCR-C2 (1:3,000) (Murari et al., 2020).

### 2.6 qPCR

Total RNAs were isolated from wild type and *clu* mutant adult flies using a Direct-zol™ RNA MiniPrep Plus Kit (Zymo Research, Irvine, CA) as per manufacturer’s recommended protocol. One microgram of total RNA was reverse transcribed using a High-Capacity cDNA Reverse Transcription Kit (Cat # 4368814, Thermo Fisher Scientific, Waltham, MA, United States) in a 20 µL reaction. cDNA was later diluted with water to 80 µL. For gene expression analysis, quantitative PCR was performed using TaqMan Gene Expression Master Mix (Thermo Fisher Scientific, Waltham, MA, United States) in a 10 µL reaction with 2 µL diluted cDNA and one of the following TaqMan probes: Dm01806850_g1 (Tom20), Dm02136274_g1 (ND-42), Dm01820354_g1 (ND-19), Dm01804649_g1 (Cyp4ac2), Dm02145551_g1 (Mil), Dm01822473_s1 (Hsp23), Dm01816546_s1 (Pepck), Dm01835343_g1 (ND-30), Dm01794109_g1 (COX4), Dm01830822_g1 (Levy) and Dm02151827_g1 (RPL32) (endogenous control) (Thermo Fisher Scientific, Waltham, MA). Fold changes were measured based on ddCt values compared to the endogenous transcript RpL32. ddCt values were converted to 2^[ddCt] to better represent the exponential nature of PCR. The average of four 2^[ddCt] values for each sample was plotted in bar graphs in GraphPad Prism. The S.E.M. and *p* values (unpaired *t*-test) were calculated using GraphPad PRISM.

### 2.7 RNA immunoprecipitation and RT-PCR

S2R+ were grown in 10 cm dishes and cross linked using a Stratalinker (Stratagene, San Diego, CA). After washing twice in cold 1X PBS cells were lysed in IP buffer [20 mM HEPES, pH 7.4; 50 mM KCl, 0.02% Triton X-100, 1% NP-40 (sub), 1 mM EDTA, 0.5 mM EGTA, 5% glycerol] supplemented with 1 mM DTT. Extract was incubated with oligo-dT magnetic beads to isolate mRNAs. mRNA was eluted and the 2nd step of the immunoprecipitation was performed using anti-Clu antibody or IgG-guinea pig as a control. Total RNA as well as Clu-bound mRNAs were isolated using RNA isolation kit (Zymo Research, Irvine, CA). RNA was stored at −80°C until further use. RT-PCR was performed using NEB one-step RT-PCR kit (New England Biolabs, Cat #E5315S) and gene specific primers (Supplementary Figure S10) for the following genes: ND-19, ND-ASHI, ND-SGDH, ND-42, ND-23, UQCR-14, UQCR-Q, CYP4AC2, COX4, COX5B, LEVY, HSP22, mRpS16, ATPsynCF6, and TOM20.

### 2.8 Mitochondrial protein extraction and BN/CN-PAGE

Mitochondria were isolated using standard differential centrifugation. In short, 50 mg of adult flies were homogenized using a 1 ml glass homogenizer with mitochondrial isolation buffer [MIB: 250 mM Sucrose, 10 mM tris-Cl (pH 7.4), 5 mM EDTA, 15 mM MgCl_2_] supplemented with protease inhibitor cocktail (Millipore Sigma, St. Louis, MO, United States) and 0.5% BSA. Extract was centrifuged twice at 1,000 g for 5 min to obtain a clear lysate. Finally, the lysate was centrifuged at 6,400 g for 15 min to obtain a pellet enriched in mitochondria. Proteins from mitochondria were extracted using mitochondrial extraction buffer (Invitrogen, Thermo Fisher Scientific, Waltham, MA, United States) and then clearing the extract by centrifuging at 20,000 g for 30 min. Protein concentrations were determined using BCA reagents (Pierce BCA protein assay Kit, Thermo Fisher Scientific, Waltham, MA, United States). About 7.5 µg of proteins were loaded into 3%–12% bis-tris gel (Thermo Fisher Scientific, Waltham, MA, United States). The inner chamber of the apparatus was filled with a 1x cathode buffer (Invitrogen, Thermo Fisher Scientific, Waltham, MA, United States) and the outside chamber was filled with 1x Native PAGE running buffer. The gel was run at 150 V for 2 h in a cold room. For CN-PAGE, the cathode buffer was replaced with 1X running buffer after 30 min of run. The gel was fixed using the suppliers’ protocol and stained with colloidal blue (Invitrogen, Thermo Fisher Scientific, Waltham, MA, United States) or silver stained using the supplier’s protocol (SilverQuest Silver Stain Kit, Invitrogen, Thermo Fisher Scientific, Waltham, MA, United States). For in-gel activity assays for mitochondrial respiratory chain complexes, CN-PAGE gels were transferred to cold water.

### 2.9 In-gel activity assays for mitochondrial respiratory chain complexes

Complex IV + I activities: CN-PAGE gels were incubated in complex IV assay buffer [50 mM sodium phosphate buffer, pH 7.4; 0.5 mg/ml diaminobenzidine tetrahydrochloride (ThermoFisher Cat # 112090250), 1 mg/ml Cytochrome c from equine heart (Millipore Sigma Cat# C2506)] at room temperature for 25–30 min. After the brown bands appeared at a desired intensity, the assay solution was replaced with water and the gel was rinsed for 1 min before adding Complex I assay buffer [2 mM Tris-Cl, pH 7.5; 0.1 mg/ml NADH (Millipore Sigma Cat # 10107735001), 2.5 mg/ml Nitrotetrazolium Blue chloride (Millipore Sigma Cat# N6876)]. Gels were incubated at room temperature for additional 15–20 min to get prominent purple bands. The reaction was stopped by adding 1/10th volume of acetic acid, then the gel was rinsed with water and imaged. To determine MRC complex levels, BN-PAGE gels were run twice with unique biological and technical replicates. For MRC complex activity assays, for CI activity, complexes were isolated two times and the assay was performed five times. For CIV activity, complexes were isolated twice and the assays was performed two times. Statistically significance for CI activity was done using GraphPad PRISM with an unpaired *t*-test. The graphs for CI and CIV were made using GraphPad PRISM.

## 3 Results

### 3.1 Tandem mass tag quantitative mass spectrometry shows loss of Clu, Sod2 and Pink1 affects the abundance of subsets of mitochondrial proteins

**FIGURE 1:**
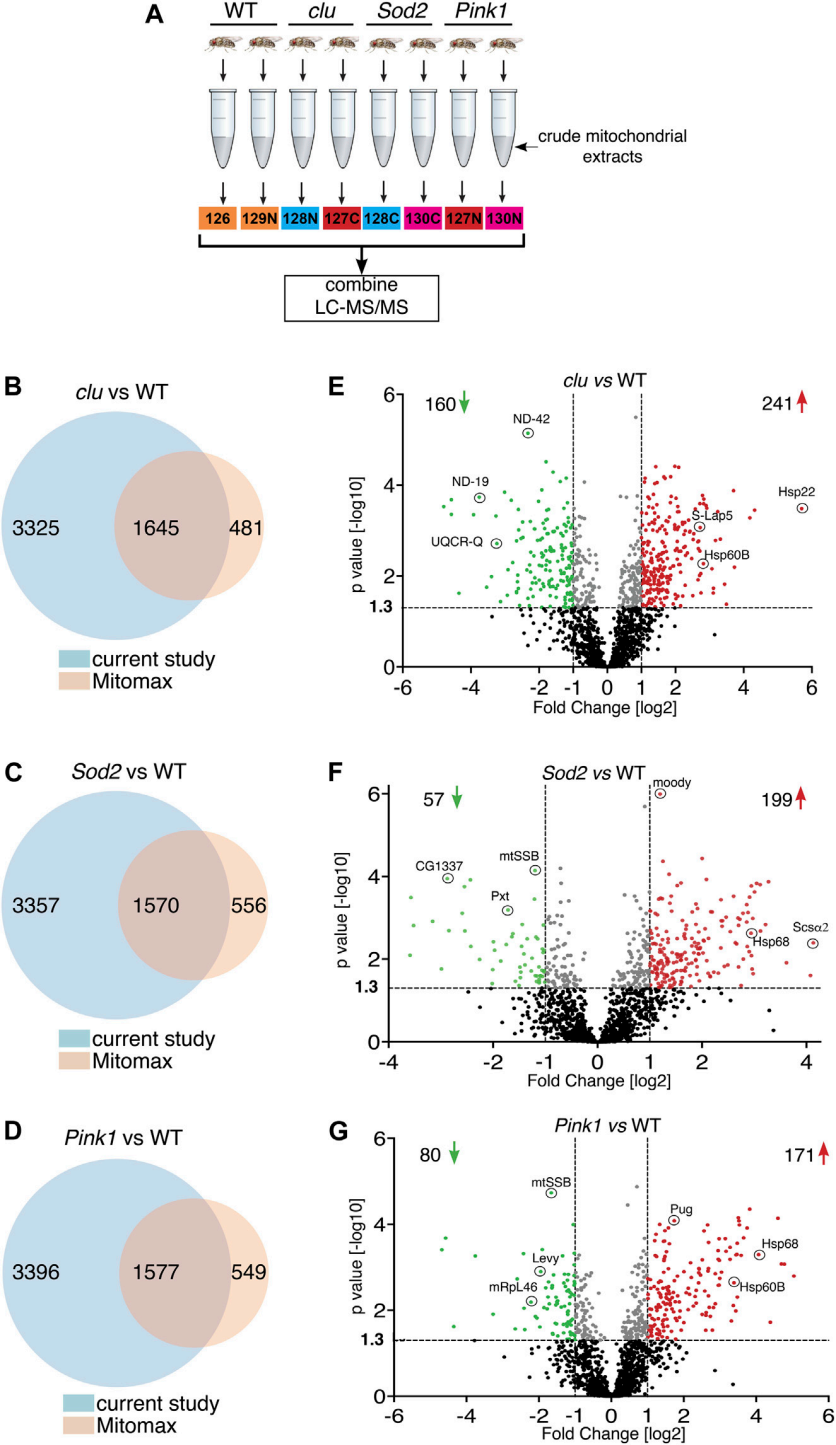
Mitochondrial protein abundance in *clu, Sod2* and *Pink1* mutants. **(A)** Schematic of the TMT mass spectrometry method for analyzing mitochondrial extract from wild type (WT), *clu*, *Sod2*, and *Pink1* mutant adults. **(B–D)** Venn diagrams comparing the number of proteins identified in *clu* **(B)**, *Sod2* **(C)** and *Pink1* **(D)** mutants vs. proteins listed in the Mitomax database. **(E–G)** Volcano plots showing the proteins identified by LC-MS/MS analysis. Proteins from *clu* **(E)**, *Sod2* **(F)** and *Pink1* **(G)** mutants were compared with WT. *p* values (-log10) and Fold Change (log2) were plotted on the *y*-axis and *x*-axis, respectively. The vertical dotted lines mark the cut-off limits for Fold Change [log2] >1 and the horizontal dotted line denotes the *p* value [−log10] = 1.3. Proteins that were significantly less abundant (i.e., Fold Change [log2] < 1 and *p* value [log10] > 1.3) are shown in green dots, whereas proteins that were significantly more abundant (i.e., Fold Change [log2] > 1 and *p* value [−log10] > 1.3) are shown in red dots. A few representative proteins from each coordinate are labeled. Proteins with statistically insignificant (*p* ≥ 0.05) abundant ratio are labeled in black dots and proteins whose abundant ratios are significant but fall within the cut-off limits for Fold Change are marked in gray dots. The number of less and more abundant proteins in each genotype are shown in the respective coordinates.

To control for the effect of general mitochondrial dysfunction on mitochondrial protein levels, and to learn more about changes to mitochondrial proteomes in different mutants, we isolated mitochondria from *clu*, *Sod2* and *Pink1* mutant adults and performed tandem mass tag (TMT) mass spectrometry using isobaric labeling (Figure 1A). From the mitochondrial extract, we identified 4,970 proteins from *clu*, 4,927 from *Sod2*, and 4,973 proteins from *Pink1* samples (Figures 1B–D; Supplementary Tables S1, S2). Mitomax is a web resource containing 2,126 mitochondrial proteins which were curated using proximity-based labeling or other mitochondrial isolation-based approaches (Yin et al., 2013; Lotz et al., 2014; Chen et al., 2015). We compared the proteins identified from our TMT mass spectrometry analysis with the Mitomax database to identify the common proteins (Figures 1B–D; Supplementary Table S3). As with the total number of proteins identified with TMT mass spectrometry, the number of identified mitochondrial proteins common between each mutant and Mitomax were similar (Figures 1B–D). To identify which proteins were statistically less or more abundant in each mutant background, we plotted protein abundance and found that while *Sod2* and *Pink1* mutants had a similar number of proteins more (red) and less (green) abundant, *clu* had double the number of less abundant mitochondrial proteins (Figures 1E–G).

**FIGURE 2:**
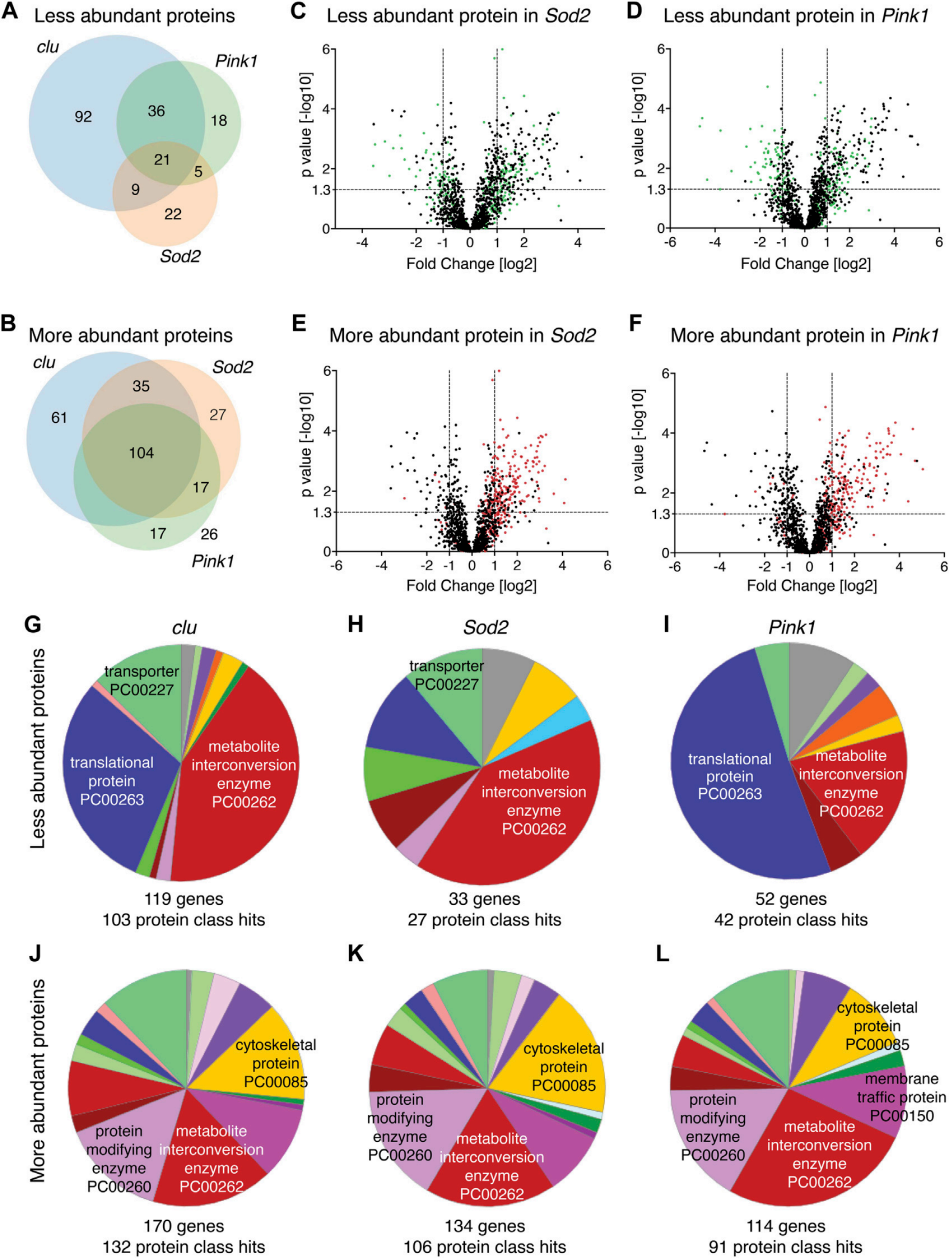
**(A,B)** Venn diagrams comparing the numbers of less abundant **(A)** and more abundant **(B)** proteins in *clu*, *Sod2* and *Pink1* mutants. **(C–F)** Volcano plots showing the distribution of proteins that were either less abundant (green dots) and more abundant (red dots) in *clu* mutants superimposed on *Sod2* **(C,E)** and *Pink1* **(D,F)** volcano plots. *p* values (−log10) and Fold Change (log2) were plotted on the *y*-axis and *x*-axis, respectively. The vertical dotted lines mark the cut-off limits for Fold Change [log2] >1 and the horizontal dotted line denotes the *p* value [−log10] = 1.3. **(G–L)** Pie charts showing PANTHER Protein Class (PC) ontology of less abundant proteins **(G–I)** and more abundant proteins **(J–L)** for *clu*, *Sod2*, and *Pink1* mutants. Numbers of genes and protein class hits are below each pie chart. Major protein class hits in each mutant were marked on pie chart slices. Names of all protein classes along with color codes and respective percent hits are in Supplementary Table S3.

Clu, Sod2 and Pink1 affect mitochondrial processes in different ways. Thus, we wanted to understand the differences between how each mutant affects mitochondrial protein abundance. First, we compared the overlap of mitochondrial protein identity for less and more abundant proteins between all three mutants (Figures 2A,B). For less abundant proteins, a greater proportion are unique in *clu* mutants [58% (92 out of 158)] compared to *Sod2* (38%, 22 out of 57) and *Pink1* (22%, 18 out of 80) mutants (Figure 2A). Only 7% (21 out of 295) are common among all three (Figure 2A). For the more abundant mitochondrial proteins, 28%, 15% and 16% are unique between *clu*, *Sod2,* and *Pink1* mutants, respectively (Figure 2B). Noticeably, the more abundant proteins common between all three mutants constitute the highest number (104) (Figure 2B).

Since loss of Clu had the greatest effect on the mitochondrial proteome, we labeled the less and more abundant mitochondrial proteins for *clu* superimposed on the Volcano plots for proteins identified from *Sod2* or *Pink1* extract (Figures 2C–F, green and red dots, respectively). The less abundant proteins in *clu* mutants are randomly distributed on Volcano plots for Sod2 and Pink1, indicating there is not a strong correlation in which proteins are less abundant between the genotypes (Figures 2C,D, green dots). In contrast, more abundant proteins identified in *clu* mutant mitochondrial extract are mostly found in the upper right quadrant of *Sod2* and *Pink1* Volcano plots, indicating they are more similar (Figures 2E,F, red dots).

### 3.2 Bioinformatic analysis indicates Clu loss decreases a specific subset of mitochondrial proteins compared to loss of Sod2 and Pink1

There are many web-based algorithms available to analyze protein datasets to identify enriched pathways (Huang da et al., 2009; Chen et al., 2013; Mi et al., 2019). By using several methods, one has an increased chance of recognizing false enrichments. To better understand the differences and similarities in mitochondrial proteomes in *clu*, *Sod2* and *Pink1* mutants, we first analyzed which protein classes were affected using PANTHER (Mi et al., 2019; Mi et al., 2021). PANTHER is a bioinformatic classification system that combines gene function, ontology and pathways to create functionally related subfamilies [Protein Class (PC)]. PANTHER analysis showed that the largest protein class for less abundant proteins in *clu* and *Sod2* mutants was metabolite interconversion enzyme (Figures 2G,H, red). In *clu* mutants, the second largest protein class was translational protein, which was the largest class in *Pink1* mutants (Figures 2G,I, dark blue). It is important to note that this analysis identified the largest number of genes and protein class hits in *clu* mutants (119 and 103, respectively) whereas many fewer were identified in *Sod2* (22 and 27, respectively) and *Pink1* (52 and 42, respectively). Thus, the pie slices for *Sod2* and *Pink1* represented fewer proteins compared to *clu* (Supplementary Table S4). PANTHER analysis for more abundant proteins in all three mutants was different. First, the abundance of each protein class was more similar between all three compared to between the less abundant proteins (Figures 2J–L vs. Figures 2G–I). The five top hit classes were metabolite interconversion enzyme, protein modifying enzyme, cytoskeletal protein and membrane traffic protein (Figures 2J–L). Second, *clu* mutants again had more gene and protein class hits (170 and 132, respectively), but *Sod2* and *Pink1* had more hits compared to less abundant proteins. Overall, this suggests that more abundant proteins are functionally more similar between the three mutants compared to less abundant proteins.

The protein class hits identified using PANTHER analysis are quite broad categories. A second way we analyzed changes to protein abundance was to use FlyEnrichr (Chen et al., 2013; Kuleshov et al., 2016). FlyEnrichr has several output options for identifying enriched pathways. We chose “biological process” which gave a more granular view of GO terms compared to the protein classes of PANTHER. FlyEnrichr analyses were performed under default conditions with the term GO Biological Process 2018 where the significance of the term was determined using combined scores (c-score = ln(adj *p*-value) ∗ z-score) in each dataset and adjusted *p*-value < 0.05 (Chen et al., 2013). Examining the less abundant proteins, 50%–70% of the top ten GO term hits for all three mutants were for mitochondrial function, including mitochondrial translation, mitochondrial gene expression, and electron transport (Table S5). As *clu* loss had the greatest number of reduced proteins, the combined score for the top ten hits was higher compared to *Pink1* and *Sod2*. This combined score reflects three different significance values, thus, the higher the score, the more likely the resulting classification is real and not a false positive. While FlyEnrichr showed *Sod2* loss also affected protein abundance for various classes of mitochondrial function, since the scores were somewhat low for each category and there were so many fewer proteins with low abundance, classification confidence was not as robust. 50% of the top ten GO terms for the proteins that are reduced in *clu* mutants were related to electron transport and oxidative phosphorylation, which was unique to Clu. There were very few mitochondrial associated categories shared for the top ten GO terms between all three mutants (one for *clu*, two for *Pink1* and none for *Sod2*) (Supplementary Table S5). GO terms for more abundant proteins were quite varied (Supplementary Table S5). We also performed GO analysis using FlyEnrichr to assess the 21 shared downregulated proteins and the 104 shared upregulated proteins. We found that shared downregulated proteins are mostly involved in mitochondrial DNA replication and DNA metabolic process, whereas the common upregulated proteins are involved in microtubule depolymerization, organelle organization, and secretary pathway regulation (Figures 2A,B; Supplementary Table S5).

**FIGURE 3:**
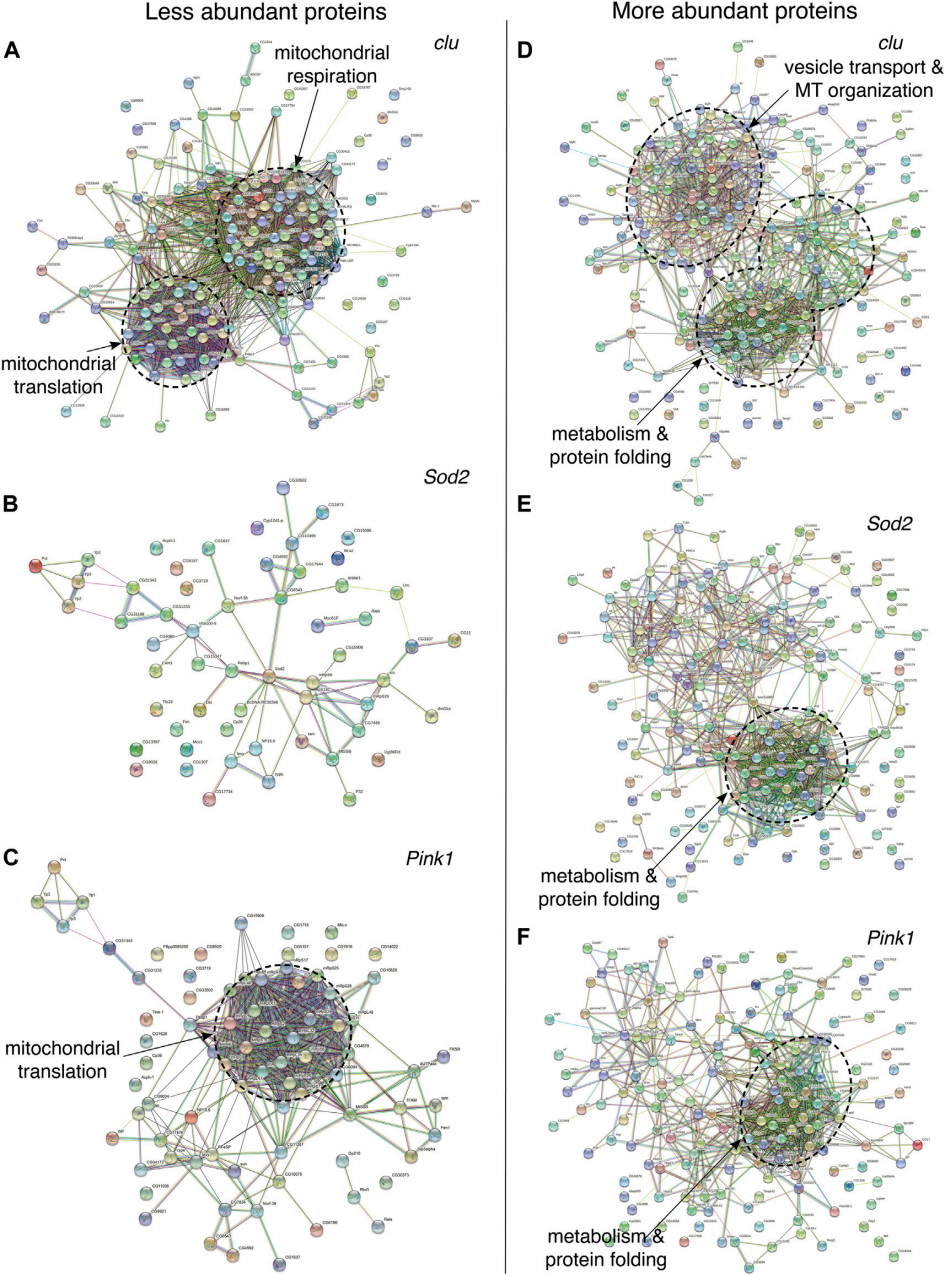
STRING protein-protein interaction (PPI) networks showing the overall clustering features of less **(A–C)** and more **(D–F)** abundant proteins in *clu*, *Sod2*, and *Pink1* mutants. Each of the major functional clusters are encircled with dashed circles and are labeled based on their compositions and biological roles. High-resolution PPI networks for each panel can be found in Supplementary Figures S1–S6 and the major candidates from each respective cluster are in Supplementary Table S6. Nodes represent proteins and edges denote interactions. The color of each node represents the nature of interactions, e.g., known or predicted interaction. A detailed description of nodes and edges can be found at STRING database (https://string-db.org/).

To further analyze which mitochondrial proteins are less and more abundant in *clu, Sod2* and *Pink1* mutants, we performed STRING analysis which groups proteins by function (Figure 3; Supplementary Figures S1–S6, Supplementary Table S6) (Szklarczyk et al., 2015). For each protein, closer proximity, with more connections and shared color, correlates with increased associated, known or predicted function (Szklarczyk et al., 2015). In *clu* mutants, the less abundant proteins fell into two major categories (Figure 3A; Supplementary Figure S1). One category was composed of proteins predominantly involved in mitochondrial respiration and electron transport chain and the second category contained proteins involved in mitochondrial translation, e.g., mitochondrial ribosomal proteins (mRPs) (Figure 3A, dashed circles, Supplementary Figure S1, Supplementary Table S6). In contrast, STRING analysis indicated that mitochondrial proteins that are down in *Sod2* mutants did not form any functional cluster (Figure 3B; Supplementary Figure S2, Supplementary Table S6). However, there was a clear functional node of less abundant proteins in *Pink1* mutants that was shared with *clu* mutants: proteins involved in mitochondrial translation (Figure 3C, dashed circle, Supplementary Figure S3, Supplementary Table S6). Thus, mitochondrial proteins related to respiration are specifically less abundant with loss of *clu* and mRPs are less abundant in *clu* and *Pink1* mutants.

STRING analysis for more abundant mitochondrial proteins indicated overlapping functional nodes between the three mutant genotypes. More abundant mitochondrial proteins in *clu* mutants generated two major categories (Figure 3D, dashed circles, Supplementary Figure S4, Supplementary Table S6). One major cluster was common between *clu*, *Sod2,* and *Pink1* mutants and was composed of proteins involved in stress response, e.g., protein folding, proteolysis, gluconeogenesis and amino acid catabolism (Figures 3D–F; Supplementary Figures S4–S6, Supplementary Table S6). *clu* mutants had the greatest number of increased proteins in amino acid catabolism, as was previously demonstrated for loss of CluH (Schatton et al., 2017). *clu* mutants also had a unique STRING cluster involved in vesicle transport and cytoskeletal organization (Figure 3D; Supplementary Figure S4, Supplementary Table S6). Thus, it appears that the stress of general mitochondrial dysfunction increases proteins involved in stress response, with *clu* absence also increasing a specific class of proteins involved in vesicle transport and the cytoskeleton.

Finally, we performed Gene Set Enrichment Analysis (GSEA) (Subramanian et al., 2005) to analyze the pathways represented by the less and more abundant mitochondrial proteins in the three mutants (Supplementary Figures S7–S9). GSEA analysis was performed using the web-based functional enrichment tool WebGestalt (WEB-based Gene SeT AnaLysis Toolkit) (Wang et al., 2017). The GSEA pathway analysis based on KEGG (Kyoto Encyclopedia of Genes and Genomes) and Reactome supported the observation from the STRING analysis (Supplementary Figures S3,S7–S9). For both analyses, genes encoding proteins belonging to the classes oxidative phosphorylation and mitochondrial translation were downregulated in *clu* and *Pink1* mutants. Metabolic pathways were also compromised in *clu* mutants. As per KEGG analysis, oxidative phosphorylation pathway proteins were reduced in *Sod2* mutants. The relative statistical significance for the KEGG analysis is more clearly indicated in Volcano plots for all three mutants (Supplementary Figure S8). Proteins that were upregulated in *clu* mutants involve amino acid metabolism as well as pathways for membrane trafficking and stress response. In *Pink1* mutants, proteins belonging to the TCA cycle and pyruvate metabolism were upregulated. Reactome Pathway analysis did not find any significant changes for *Sod2* mutants (Supplementary Figure S9). In all of the above cases, FDR ≤0.05 was the cut-off limit.

### 3.3 Complex I proteins are reduced in *clu* mutants compared to *Sod2* and *Pink1*

**FIGURE 4:**
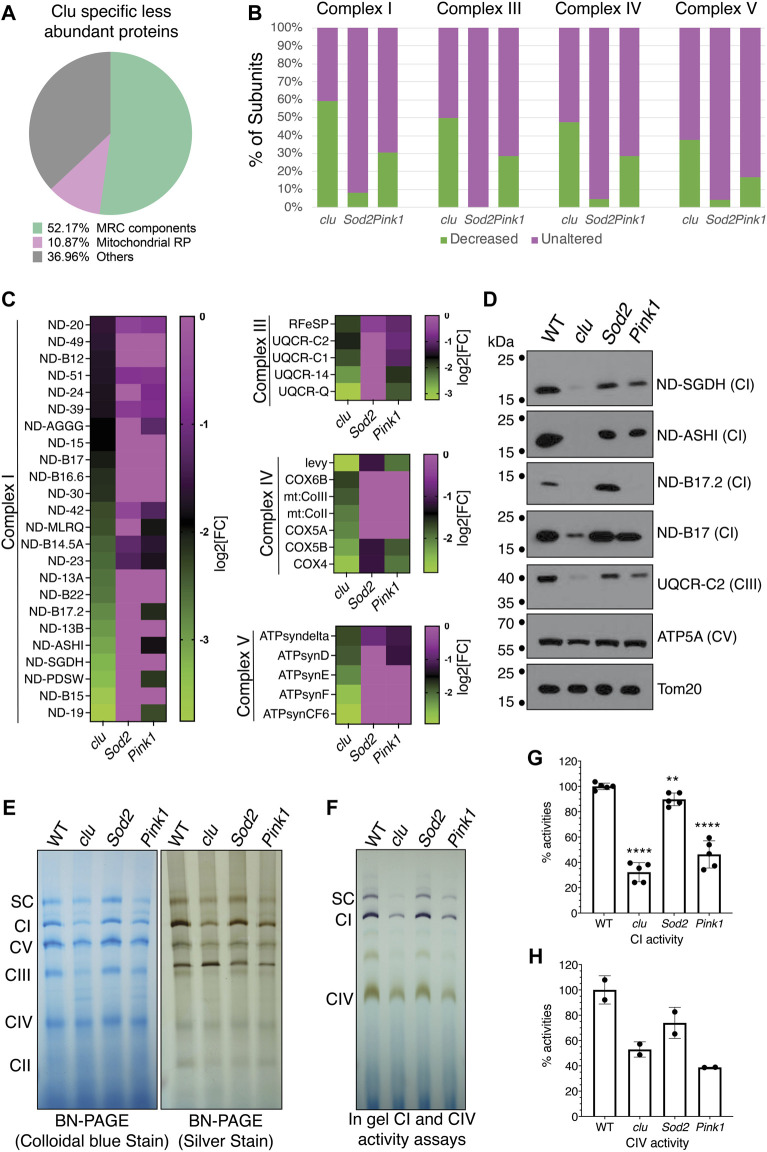
Mitochondrial respiratory chain proteins are greatly decreased in *clu* mutants. **(A)** A pie chart showing *clu* specific less abundant proteins in different categories as percentage of the whole [total = 92 (Figure 2A)]: Mitochondrial Respiratory Chain (MRC) components, Mitochondrial Ribosomal Proteins (Mitochondrial RP) and Others. **(B)** Bar graph showing the number of subunits, in percentages, from each entire MRC are either decreased (in green) or remain unaltered (in magenta). **(C)** Heat maps showing the levels of affected individual MRC proteins of different complexes in *clu*, *Sod2*, and *Pink1* mutants. The proteins chosen are decreased in *clu* mutants. Color coded scale bars (green: less abundant and magenta: unaltered) are representing the log2[Fold Change] of individual protein in each mutant compared to wild type. **(D)** Western blots showing the levels of representative proteins in *clu*, *Sod2*, and *Pink1*. Tom20 was used as a loading control. **(E)** Proteins from isolated mitochondria were run to determine the levels of native MRC complexes on a BN-PAGE (left panel, stained with colloidal blue, right panel, stained with SilverQuest Silver Staining Kit). **(F)** In-gel activity assays. Dark purple bands indicate CI activity and brown bands indicate CIV activity. **(G,H)** The activities from CI **(G)** and CIV **(H)** were determined by measuring band intensity of the respective complexes using Fiji software. Band intensities from three gels (*n* = 3) for CI and two gels (*n* = 2) for CIV were used for the measurements. Error bars: S.E.M. calculated in GraphPad-PRISM software. *p* values were calculated in GraphPad-PRISM software using an unpaired *t*-test and each mutant was compared to the wildtype control. Statistical significance = *p* < 0.0001 (****) and *p* < 0.003 (**).

Our STRING analysis indicated that mitochondrial proteins related to respiration were specifically less abundant with *clu* loss (Figure 3A). The largest category in this *clu*-specific STRING cluster was mitochondrial respiratory chain (MRC) components (Figure 4A, green). Analyzing a breakdown of MRC proteins that were less abundant for Complex I (CI), Complex III (CIII), Complex IV (CIV) and Complex V (CV) in each mutant background indicated that *Sod2* loss had little effect on MRC proteins, *clu* loss had the strongest effect, and *Pink1* loss was intermediate (Figure 4B, green). We further compared the relative changes of specific MRC proteins using a heat map (Figure 4C). For each Complex, we compared the MRC proteins that are less abundant in *clu* mutants (Figure 4B, green) to their abundance in *Sod2* and *Pink1*. For all the proteins that are less abundant in *clu* mutants, *Sod2* mutants showed little change whereas *Pink1* mutants had an intermediate amount of difference for the MRC candidates (Figure 4C).

To validate our findings, we performed Western blots for representative proteins from CI, CIII, CIV and CV (Figure 4D). The western blot analysis confirmed that the levels of various subunits were greatly reduced in *clu* mutants with no effect in *Sod2* and with *Pink1* affecting some proteins. We used Tom20 as a loading control because our mass spectrometry data indicated no change in the level of Tom20 between the three genotypes and wild type (Supplementary Table S2). As the majority of MRC proteins was greatly reduced in *clu* mutants, we wanted to verify the integrity and activity of the respiratory complexes. To analyze the level of all MRCs, we ran mitochondrial extracts on Blue Native PAGE (BN-PAGE) and Clear Native PAGE (CN-PAGE) (Figure 4E). The band intensity was reduced for most complexes in *clu* mutants. Complexes isolated from *Sod2* mutants had similar band intensity compared to wild type, whereas complexes from *Pink1* mutants were only somewhat decreased in agreement with our heatmap analysis and western blots. Next, we assessed the activity of CI, CIV and the super complex (SC) using in-gel activity assays (Figures 4F–H). Activities of CI and CIV were highly reduced in *clu* mutants as compared to wild type and *Sod2* (Figures 4F–H). CI and CIV activity were also significantly reduced for *Pink1* mutants. Thus, respiratory complex integrity and activity was reduced for *clu* and *Pink1*, whereas *Sod2* was mostly unaffected.

### 3.4 Protein low abundance is not due to decreased transcript levels

**FIGURE 5:**
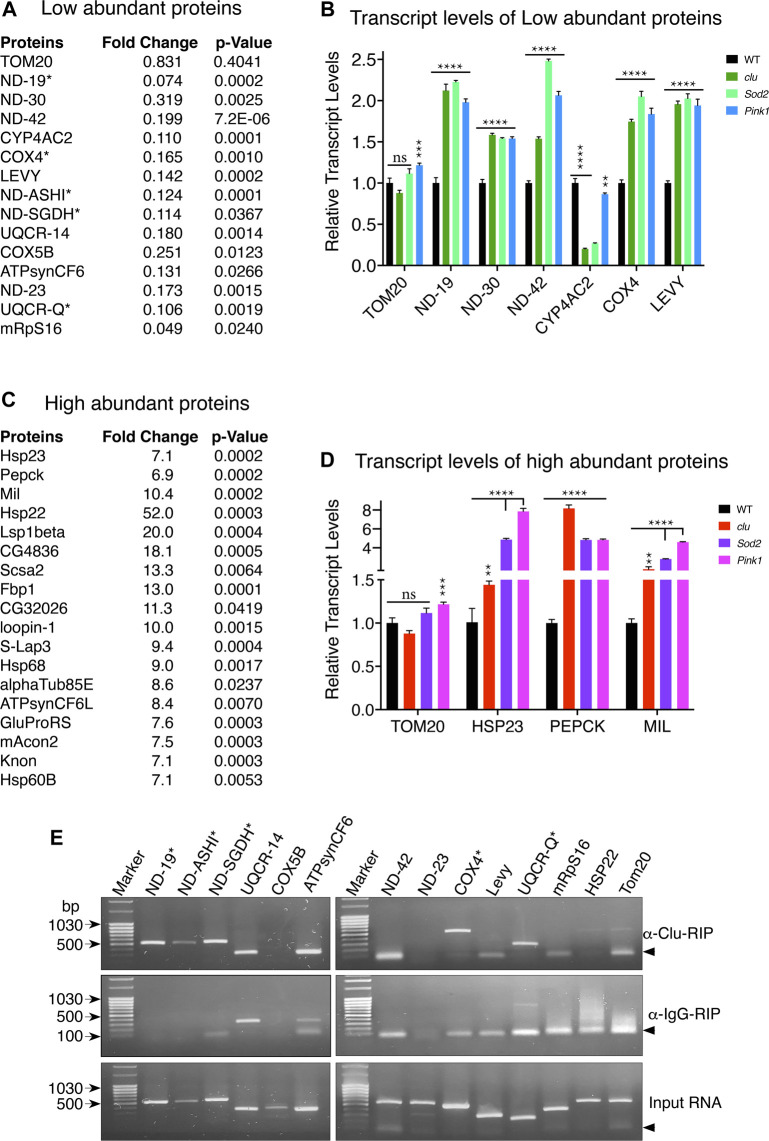
Loss of *clu*, *Sod2*, and *Pink1* increase transcript levels for candidate mitochondrial proteins. **(A,C)** Representative mitochondrial proteins with respective fold changes and *p* values for less **(A)** and more **(C)** abundant proteins in *clu* mutants. The transcripts of proteins marked with asterisks bind Clu. **(B,D)** Bar graphs showing the relative transcript levels of selected less **(B)** and more **(D)** abundant proteins in *clu*, *Sod2*, and *Pink1* mutants. **(E)** RT-PCR after Clu immunoprecipitation (RIP). anti-IgG and input are the two controls. Candidates marked with asterisks bind Clu. Arrowheads show the location of nonspecific primer-primer dimers. **(B,D)** Error bars: S.E.M. calculated in GraphPad-PRISM software. *p*-values were calculated in GraphPad-PRISM software using an unpaired *t*-test and each mutant was compared to the wildtype control. Statistical significance = *p* < 0.000001 (****), *p* < 0.00001 (***), *p* < 0.005 (**), ns = non-significant.

Changes in protein levels can be due to several reasons. For example, protein degradation can be up or downregulated, mRNAs can become stabilized or destabilized or transcription can be up or downregulated. Since *clu* loss in particular affected MRC abundance and activity, and *clu* loss specifically decreased proteins involved in mitochondrial respiration (Figure 3A), we focused on MRC proteins to determine whether the reduced amount present in TMT mass spec analysis was due to decreased transcript level (Figure 5A). To do this, we performed quantitative PCR analysis to check the relative abundance of several candidate transcripts (Figure 5B). We chose six proteins less abundant in *clu* (ND-19, ND-30, ND-42, CYP4AC2, COX4, and LEVY) and one that showed no change (TOM20) between the mutants and wild type (Figure 5B; Supplementary Table S2). As expected, TOM20 transcript levels were mostly consistent between the three genotypes. Surprisingly, for steady state levels of transcript, loss of any of the three mutants significantly increased transcript levels. This was regardless of how abundant the protein was (Figure 4C). The only exception among the chosen candidates was CYP4AC2, a protein that may be involved in insect hormone biosynthesis, which was decreased in *clu* and *Sod2* mutants, but not *Pink1*. Conversely, we were interested in steady state transcript levels for a small number of high abundance candidates and chose candidates identified in *clu* mutants (Figure 5C). We analyzed HSP23, PEPCK, and MIL (Figure 5D). As with less abundant proteins, all three transcripts were increased in all three mutants. These data suggest that the steady state levels of transcript are not related to lower protein abundance as measured by TMT mass spec and that general mitochondrial dysfunction causes increased transcript levels for our candidates identified in both low and high abundant *clu* proteins.

### 3.5 Clu binds mRNAs encoding a subset of mitochondrial respiratory chain proteins

Since Clu/CLUH binds nucleus-encoded mRNA, we analyzed if mitochondrial proteins that are less abundant in *clu* mutants associate with Clu, focusing on MRC transcripts. To do this we performed immunoprecipitation using anti-Clu or control IgG Guinea Pig antibodies followed by targeted RT-PCR against representative candidates across the respiratory chain components. We also used control RNA as a template for a positive control. We tested whether RT-PCR recognized products for the following mRNAs: ND-19, ND-ASHI, ND-SGDH, ND-42, ND-23, UQCR-14, UQCR-Q, CYP4AC2, COX4, COX5B, LEVY, HSP22, mRpS16, ATPsynCF6, and TOM20 (Figure 5E). After comparing with products from IgG-Guinea pig control IP, we found a subset of the low abundance candidates positively identified in the pellet following Clu immunoprecipitation and RT-PCR: ND-19, ND-ASHI, ND-SGDH, COX4 and UQCR-Q (Figure 5E, asterisks). Six low abundance candidates were not identified with RT-PCR: ND-42, UQCR-14, Levy, COX5B, ATPsynCF6, and mRpS16. Furthermore, the most significantly highly abundant candidate, Hsp22, was also not identified. These experiments clearly suggest that Clu binds selected mRNAs encoding mitochondrial respiratory chain proteins.

## 4 Discussion

### 4.1 The number of proteins in mitochondrial proteomes varies depending on the type of analysis

Mitochondria were once viewed as static organelles whose main function was to provide ATP. Research from the past decades has established that these organelles are highly dynamic and change shape, numbers and activity to suit the needs of different tissues and cell types (Chan 2006; Youle and van der Bliek 2012). In addition, mitochondrial defects including generalized oxidative damage, mtDNA mutations and single nDNA mutations contribute to many diseases (Zeviani et al., 2003). As our understanding has increased, so has the number of proteins associated with mitochondrial function (Area-Gomez and Schon 2014). mtDNA encodes only 13 proteins, thus mitochondria rely on nucleus-encoded genes for all functions, including mitoribosome assembly and function, fission and fusion, movement along the cytoskeleton and many metabolic processes. Mitochondria have four compartments: the outer mitochondrial membrane, the intermembrane space, the inner mitochondrial membrane and the matrix. Thus, the mitochondrial proteome can be defined and compartmentalized in several ways. In addition, the outer mitochondrial membrane associates with large classes of proteins, including those involved in transport and other peripherally associated functions. Researchers obtain mitochondrial proteomic data from whole tissue/cell extract and from isolated mitochondrial extract which contributes to the variability of proteins. In this study, we used crude mitochondrial extract from Drosophila adult mutants 1–4 days old. This ensured we would capture not just proteins present in the four mitochondrial compartments, but also mitochondrially associated cytoskeletal elements and other peripheral proteins. A caveat is that potentially spurious proteins would also be present as evident from our TMT mass spectrometry analysis.

There are several databases that are valuable resources for analyzing mitochondrial proteomes in different organisms (Sardiello et al., 2003; Lott et al., 2013; Chen et al., 2015; Smith and Robinson 2019; Rath et al., 2021). These databases are periodically updated using more advanced experimental and computational inputs. For example, the mammalian mitochondrial database, MitoCarta, has been updated with mitochondrial compartment and pathway specific information from mitochondria isolated from fourteen tissue samples (Rath et al., 2021). In Drosophila, MitoMax, a newly developed mitochondrial protein database, provides a comprehensive view of the Drosophila mitochondrial proteome incorporating other databases like MitoDrome, MitoMiner, MitoCarta as well as from mitochondrial isolation-based proteomic analysis. These databases highlight the complexity and dynamic nature of the mitochondrial proteome.

### 4.2 Single gene mutations that affect mitochondrial function differentially affect the mitochondrial proteome

Clu, Sod2, and Pink1 are critical for mitochondria, but function in very different ways. Loss of all three proteins reduces ATP and increases mitochondrial oxidative damage. Drosophila *clu*, *Sod2* and *Pink1* mutants have reduced lifespan, although *Pink1* mutants live substantially longer than the other two (Duttaroy et al., 2003; Clark et al., 2006; Park et al., 2006; Sen et al., 2013). Clu/Cluh are essential ribonucleoproteins associated with ribosomal proteins that may regulate mRNAs for co-translational import into mitochondria (Sen and Cox 2016; Vardi-Oknin and Arava 2019; Hemono et al., 2022). Clu forms large, dynamic cytoplasmic particles that are sensitive to stress (Cox and Spradling 2009; Sheard et al., 2020; Sheard and Cox 2021). SOD2 is an important free radical scavenger located in the mitochondrial matrix. SOD2 converts damaging superoxide into hydrogen peroxide (Weisiger and Fridovich 1973). Finally, Pink1 is involved in mitophagy (Pickles et al., 2018). Pink1 also binds mRNAs encoding MRCs mRNAs at the mitochondrial outer membrane and thus may function in co-translational import (Gehrke et al., 2015). Thus, while all three proteins are crucial for mitochondrial function and ultimately cause similar damage, they perform very different roles in the cell.

Proteomic analysis is available for mouse *CluH KO* and Drosophila *Pink1* mutants (Vincow et al., 2013; Schatton et al., 2017). Proteins isolated from whole liver extract from E18.5 *CluH* knockout mice were compared to that of E18.5 wild type mice using LC/MS, then compared with a control P1 mouse liver fully labeled with heavy [^13^C_6_] lysine as a SILAC control (Schatton et al., 2017). Using this technique, Schatton et al. identified 525 proteins that mapped to mitochondria, 40% of which were reduced in the *CluH* knockout mouse liver extracts with ≥1.5 fold change. They showed that *CluH KO* mouse livers have reduced protein levels for mitochondrial proteins involved in metabolic pathways and oxidative phosphorylation. This analysis did not find any significant increase in mitochondrial proteins or transcripts levels. We also found *clu* mutants have significantly fewer mitochondrial proteins involved in mitochondrial respiration and translation. In fact, *clu* mutants had the largest number of less and more abundant mitochondrial proteins. We also found that all MRC proteins are less abundant. As with *CluH* knockout mice, we also saw reduced respiratory complex activity. Using STRING analysis, we identified distinct classes of mitochondrial proteins that were more abundant, including proteins involved in stress such as proteins involved in metabolism, protein folding and amino acid catabolism. This stress class was shared by *Sod2* and *Pink1* mutants. Finally, *clu* mutants had more abundant cytoskeletal proteins and vesicle transport proteins, although all three mutants showed some association with increased cytoskeletal elements by GO term Biological Process analysis. Cells mutant for *clu*, *CluH*, the Arabidopsis ortholog *FRIENDLY MITOCHONDRIA*, and the Dictyostelium ortholog *CluA*, have highly mislocalized and clumped mitochondria which could be one explanation (Zhu et al., 1997; Fields et al., 1998; Cox and Spradling 2009; El Zawily et al., 2014; Gao et al., 2014). However, *Pink1* and *Sod2* mutants also have mislocalized mitochondria, but do not increase the levels of these protein classes (Sen et al., 2015; Sheard et al., 2020). Clu has been shown to have a role in muscle integrity, thus perhaps it has a greater effect on cytoskeletal elements (Wang et al., 2016).

To determine whether Pink1 is involved in mitophagy, Vincow et al. (2013) used SILAC labeling to identify whether proteins have longer half-lives in *Pink1* mutant heads, potentially due to impeded mitophagy. To do this, they fed mutant or control flies heavy D3-leucine five to 10 days then performed mass spectrometry (Vincow et al., 2013). From this analysis, Vincow et al. (2019) showed that only MRC proteins had longer half-lives, compared to non-MRC proteins, and that of the 45 identified MRC proteins, this effect was primarily due to those that are in the membrane. We found that loss of Pink1 resulted in fewer proteins involved in mitochondrial translation, such as mitochondrial ribosomal proteins, which was similar to *clu* mutants. In addition, *Pink1* mutant flies appeared to have more abundant proteins related to stress, namely, heat shock proteins and proteins involved in metabolism and amino acid catabolism. Vincow et al. (2019) also examined the half-life of mitochondrial proteins in a transheterozygote *Sod2* mutant and found no difference in protein half-life compared to control. We used a *Sod2* null mutant which only lives 1 day (Duttaroy et al., 2003). For less abundant mitochondrial proteins, Sod2 showed the least correlation with specific pathways or GO terms. However, these mutants had more abundant proteins involved in stress and metabolism.

The less abundant proteins shared between all three mutants were overwhelmingly represented by GO terms involved in mitochondrial processes (8/10). Of the top ten GO terms, five were clearly mitochondrial and three were likely mitochondrial. An additional term was axonal transport of mitochondria (GO:0019896). One GO term appears to potentially be a false positive since it is tightly associated with humoral immune response, although mitochondrial diseases are associated with disruptions to immune response (Kapnick et al., 2018). In general, the common less abundant proteins support that the resulting mitochondrial damage from loss of each gene causes disruption to mitochondrial biosynthetic processes, such as mtDNA replication and maintenance and biomolecule synthesis.

Perhaps more surprising were the two shared classes of proteins that were more abundant. It is possible that these two classes are false positives, however, there are potential reasons why they would be more abundant. Of the top ten shared GO terms, 4/10 were microtubule organization and 4/10 were synaptic function. Control of microtubule polymerization would likely affect mitochondrial movement and positioning in the cell. General mitochondrial damage caused by a variety of insults is well known to cause mitochondrial clustering in various cell types (Al-Mehdi et al., 2012; Agarwal and Ganesh 2020; Sheard and Cox 2021). Whether this mechanism protects the cell from increasing oxidative damage or is a result of damage is not known. The second more abundant class appeared to be related to synaptic function. Since our sample was whole body, it would include all central and peripheral nervous system tissues. Since loss of mitochondrial function likely plays a role in neurodegenerative disease, increased mitochondria-associated proteins involved in synaptic function could be upregulated in response to decreased mitochondrial function in order to support synapses (Verstreken et al., 2005; Cai and Tammineni 2017).

There exist many mitochondrial diseases that arise from single nDNA gene mutations (Zeviani et al., 2003; Area-Gomez and Schon 2014). To better understand disease etiology, researchers try to understand the molecular mechanisms of each protein in order to shed light on how lack of the process could cause mitochondrial dysfunction and disease. However, studies often do not examine the overall effect particular mutations have on the mitochondrial proteome. Given that mitochondria are an important metabolic nexus in the cell, it can be difficult to parse whether resulting tissue decline is due directly to loss of a single protein or is a secondary effect from general mitochondrial damage. This work suggests that both things occur. *clu*, *Sod2*, and *Pink1* mutant flies appear to have more abundant proteins related to stress supporting a general response to mitochondrial damage that is shared by all three mutations. Since *clu* (and possibly *Pink1*) may function in co-translational import, the less abundant proteins could be directly related to loss of co-translational import. As our proteomic analysis looks only at the endpoint of protein abundance, it does not address dynamic changes to transcription or translation. Future studies comparing global transcript changes and mRNA stability could shed light on the dynamism of the mitochondrial proteome with loss of these three genes.

## Data Availability

All the data used to draw the conclusions in this manuscript are available in the main figures and supporting information. The mass spectrometry proteomics data have been deposited to the ProteomeXchange Consortium via the PRIDE partner repository with the dataset identifier PXD036616 (Perez-Riverol et al., 2022).

## Abbreviations

Clu, Clueless; CLUH, Clustered mitochondria homolog; CI, Complex I; CII, Complex II; CIII, Complex III; CIV, Complex IV; CV, Complex V; GO, Gene ontology; MRC, mitochondrial respiratory chain; PANTHER, Protein Annotation through Evolutionary Relationship; PINK1, PTEN-induced putative kinase 1; STRING, Search Tool for the Retrieval of Interacting Genes/Proteins; Sod2, Superoxide Dismutase 2; TMT, tandem mass tag.
