# Supplementary material for: Loss of Drosophila Clueless differentially affects the mitochondrial proteome compared to loss of Sod2 and Pink1
Source: Front Physiol. 2022 Oct 26;13:1004099. doi: 10.3389/fphys.2022.1004099 (PMC9644064; doi:10.3389/fphys.2022.1004099)
Supplement: Supplementary file 7 [file Image1.pdf]

Figure S1

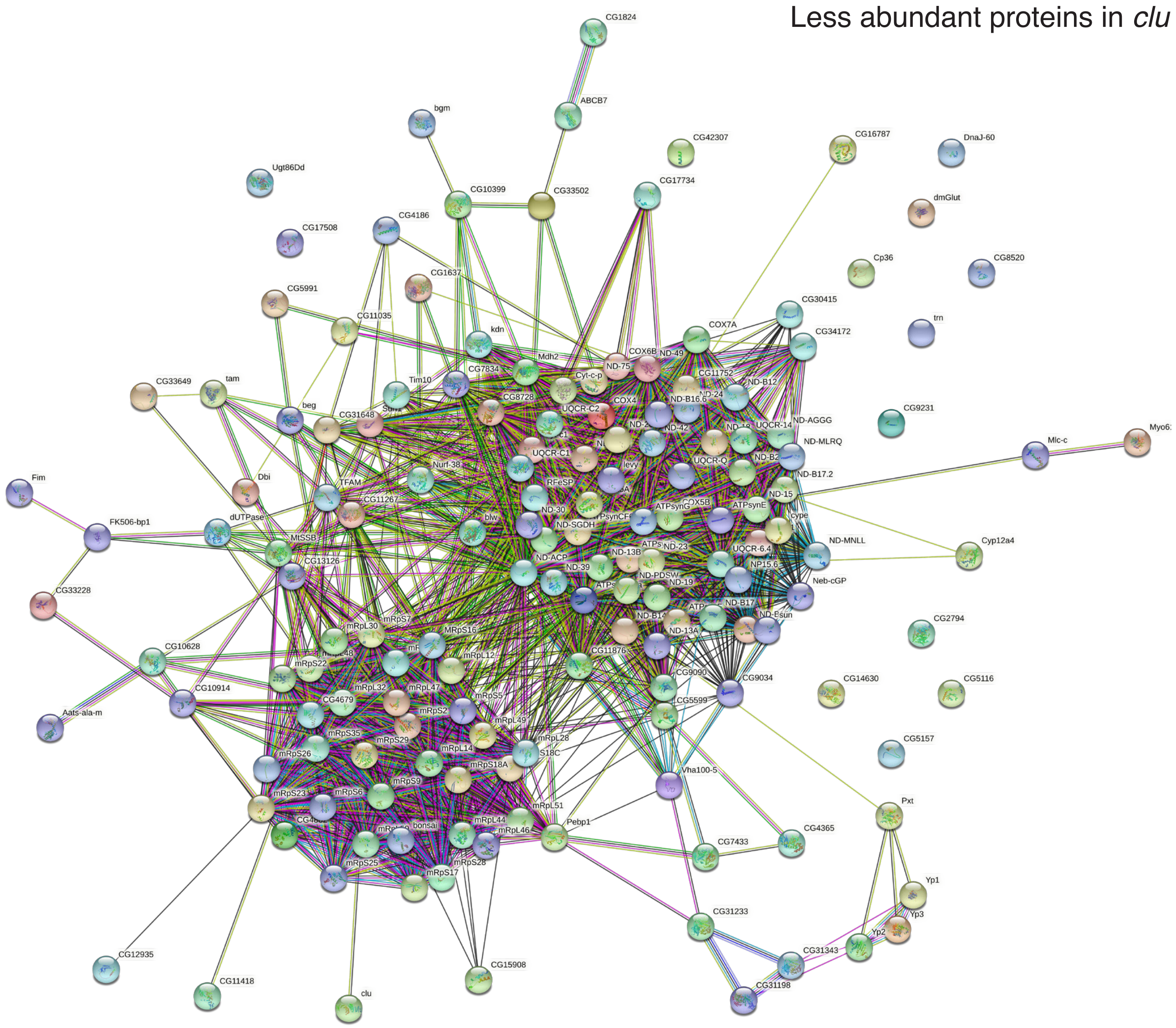

Supplemental Figure S1: The STRING protein-protein interaction (PPI) network showing the functional clustering features of less abundant proteins in *clu* mutants identified with TMT mass spectrometry analysis. The nodes (circles) represent proteins and edges (lines) denote interactions. The color of each nodes represents the nature of the interactions, e.g., known or predicted interaction. A detailed description of the nodes and the edges can be found at the STRING data base (http://version10.string-db.org/help/getting_started/).

Figure S2

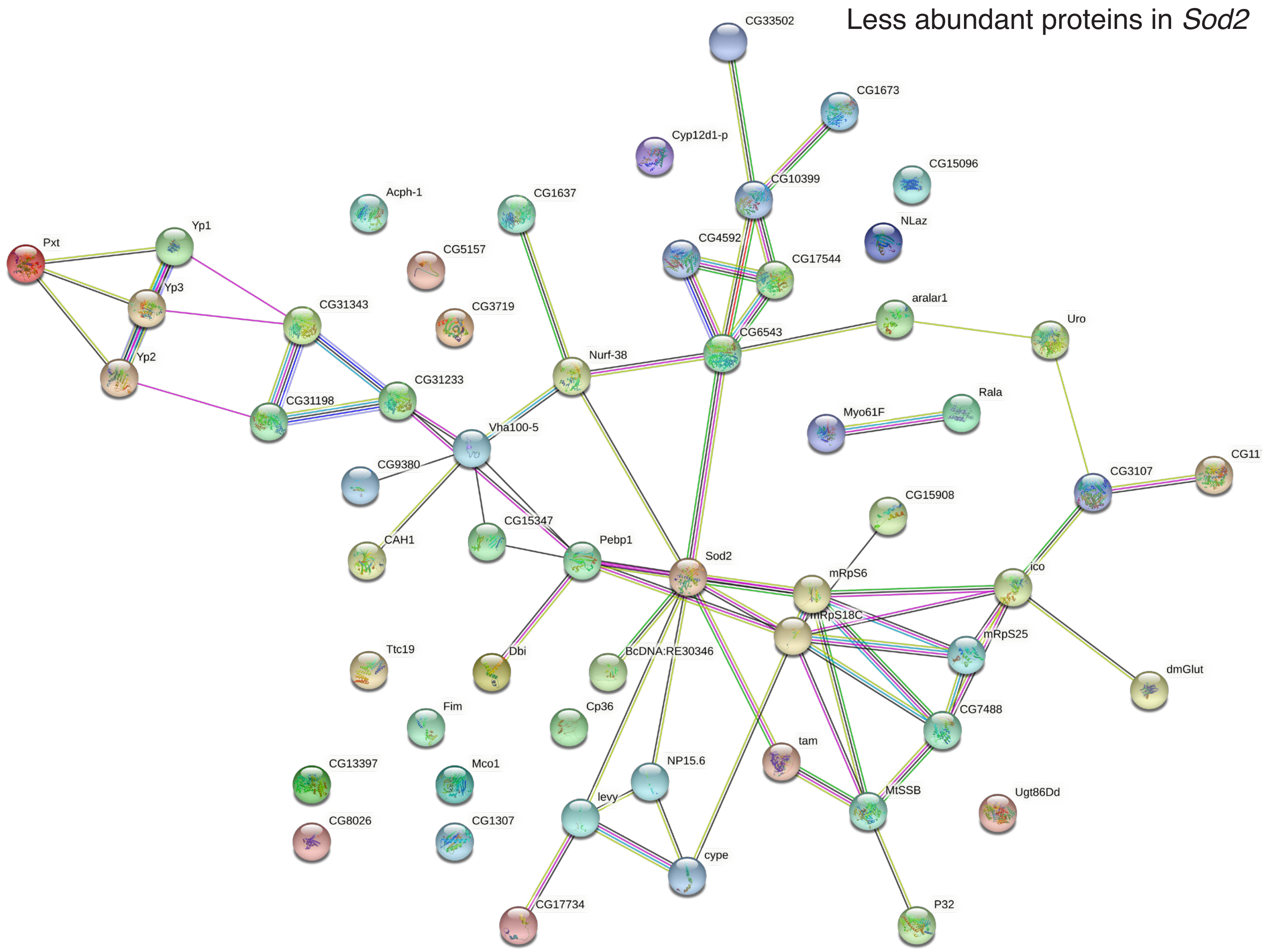

Supplemental Figure S2: The STRING protein-protein interaction (PPI) network showing the functional clustering features of less abundant proteins in *Sod2* mutants identified with TMT mass spectrometry analysis. The nodes (circles) represent proteins and edges (lines) denote interactions. The color of each nodes represents the nature of the interactions, e.g., known or predicted interaction. A detailed description of the nodes and the edges can be found at the STRING data base (http://version10.string-db.org/help/getting_started/).

Figure S3

Less abundant proteins in *Pink1*

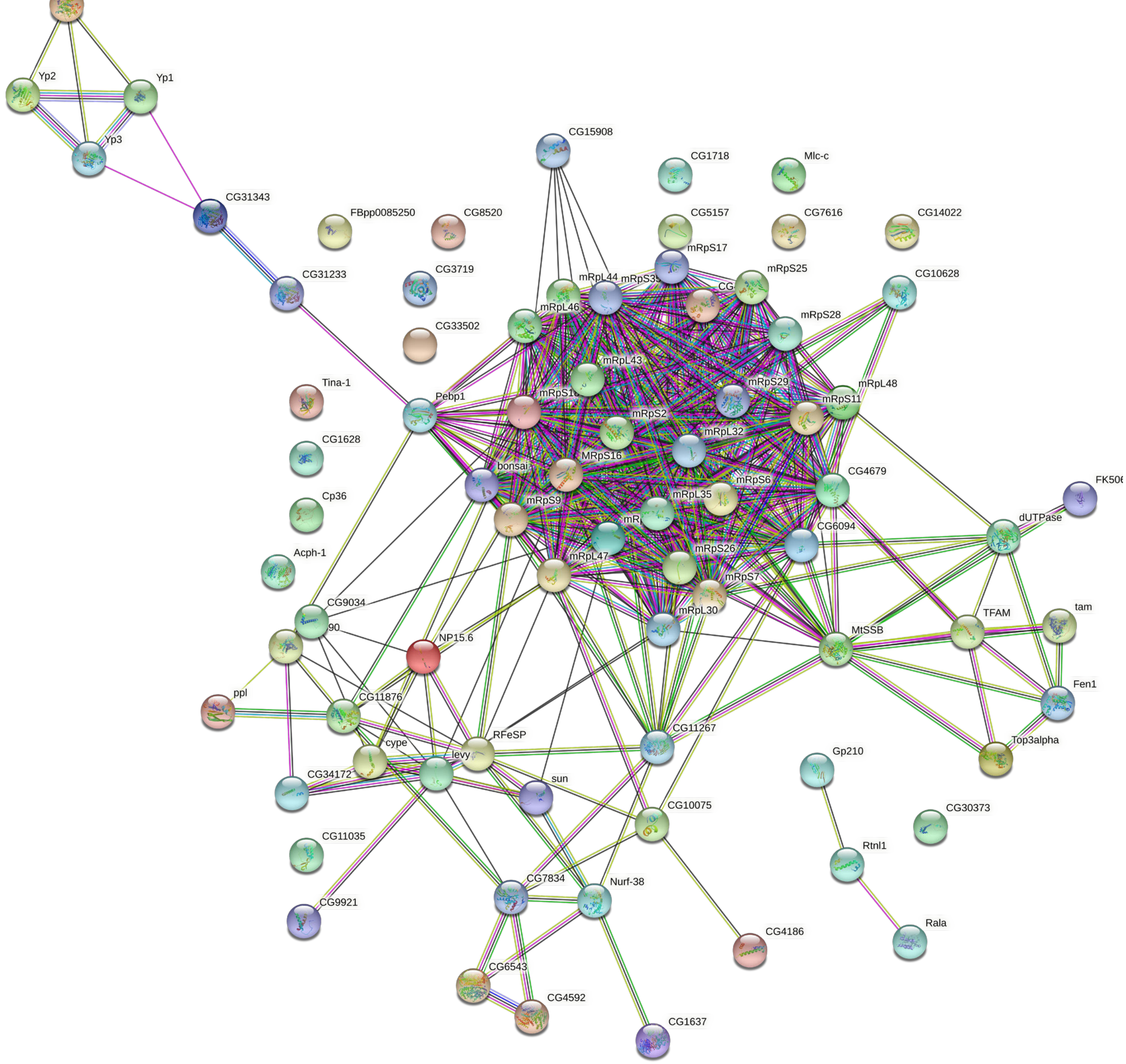

Supplemental Figure S3: The STRING protein-protein interaction (PPI) network showing the functional clustering features of less abundant proteins in *Pink1* mutants identified with TMT mass spectrometry analysis. The nodes (circles) represent proteins and edges (lines) denote interactions. The color of each nodes represents the nature of the interactions, e.g., known or predicted interaction. A detailed description of the nodes and the edges can be found at the STRING data base (http://version10.string-db.org/help/getting_started/).

Figure S4

More abundant proteins in *clu*

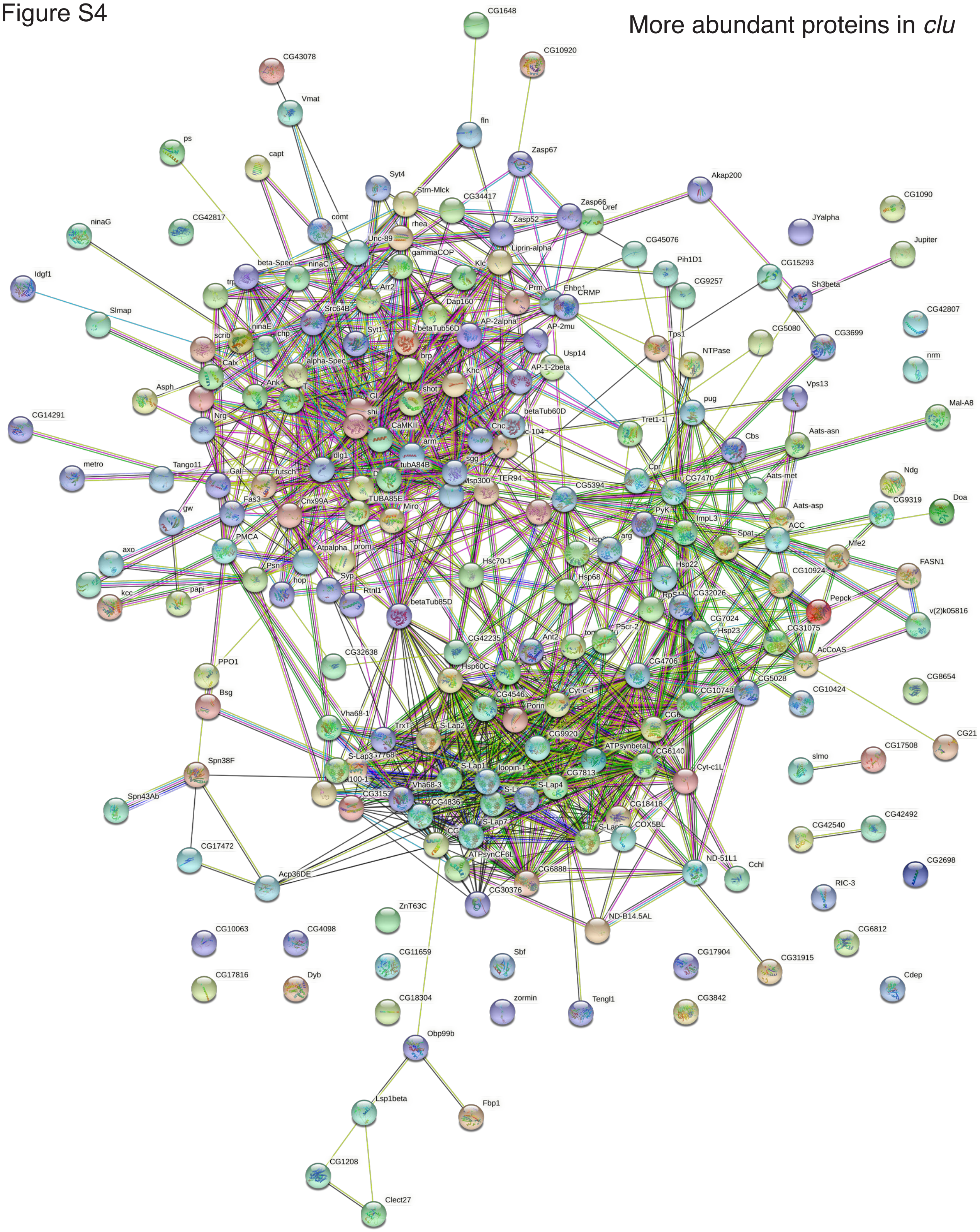

Supplemental Figure S4: The STRING protein-protein interaction (PPI) network showing the functional clustering features of more abundant proteins in *clu* mutants identified with TMT mass spectrometry analysis. The nodes (circles) represent proteins and edges (lines) denote interactions. The color of each nodes represents the nature of the interactions, e.g., known or predicted interaction. A detailed description of the nodes and the edges can be found at the STRING data base (http://version10.string-db.org/help/getting_started/).

Figure S5

More abundant proteins in *Sod2*

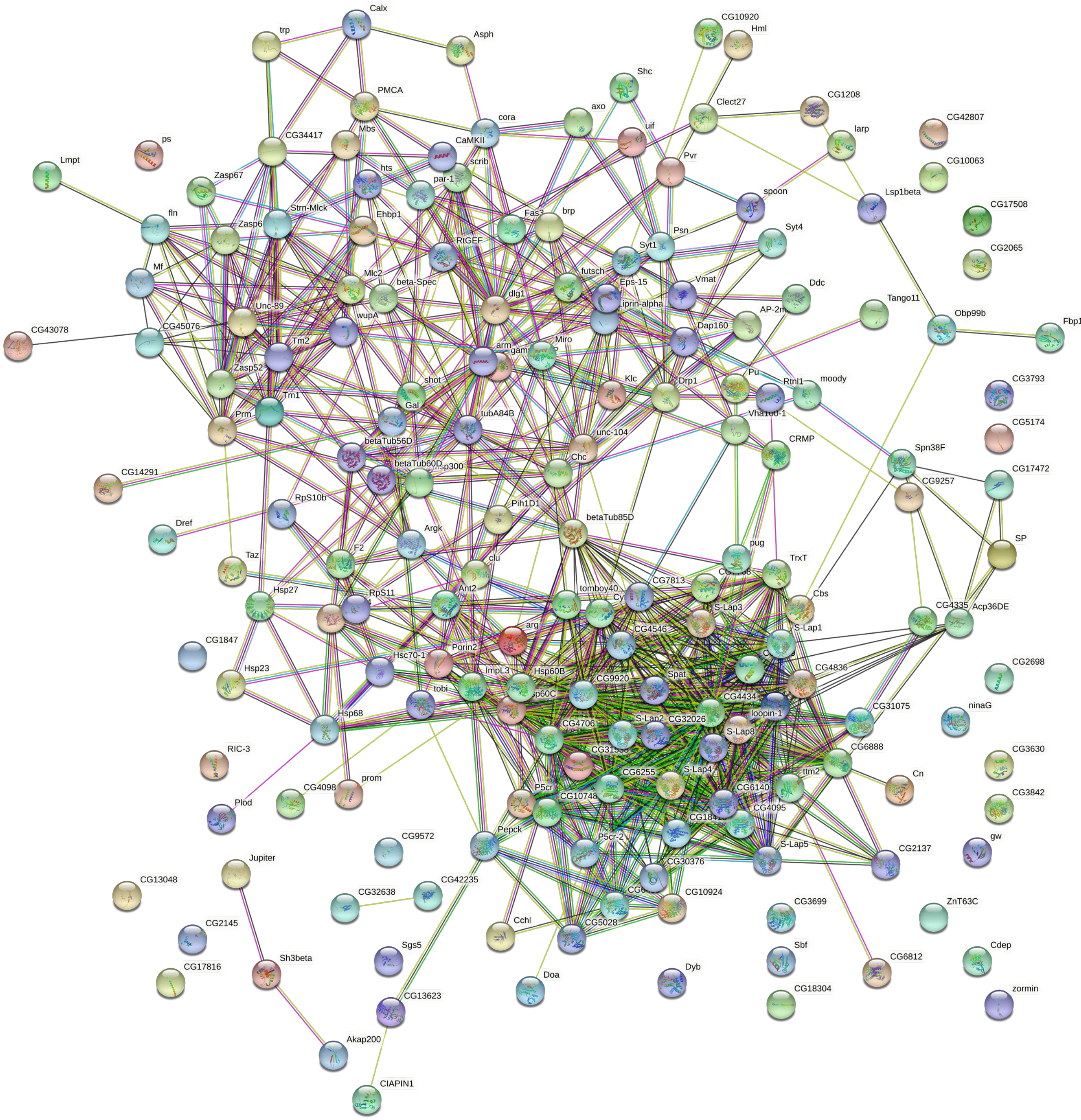

Supplemental Figure S5: The STRING protein-protein interaction (PPI) network showing the functional clustering features of more abundant proteins in *Sod2* mutants identified with TMT mass spectrometry analysis. The nodes (circles) represent proteins and edges (lines) denote interactions. The color of each nodes represents the nature of the interactions, e.g., known or predicted interaction. A detailed description of the nodes and the edges can be found at the STRING data base (http://version10.string-db.org/help/getting_started/).

Figure S6

More abundant proteins in *Pink1*

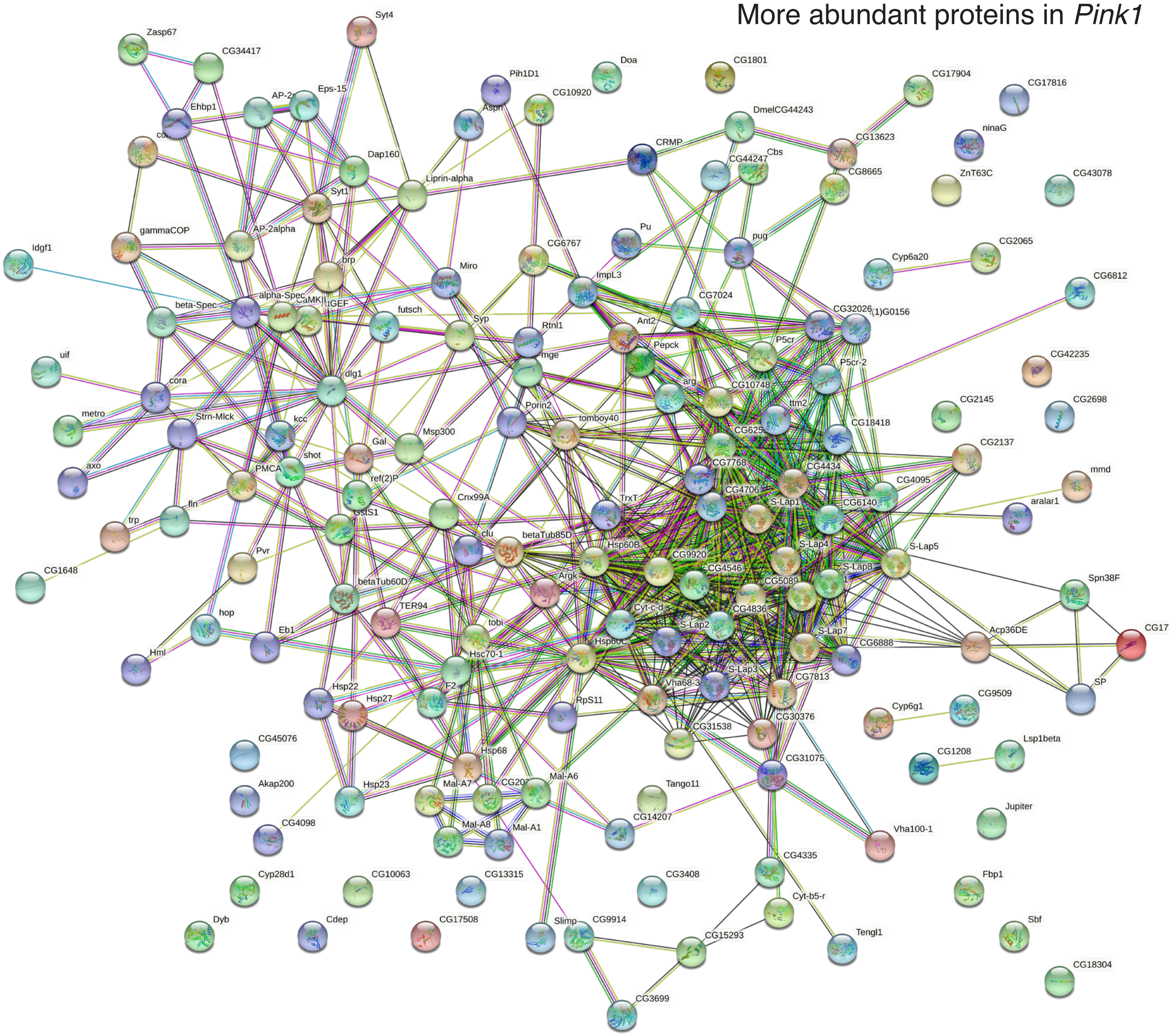

Supplemental Figure S6: The STRING protein-protein interaction (PPI) network showing the functional clustering features of more abundant proteins in *Pink1* mutants identified with TMT mass spectrometry analysis. The nodes (circles) represent proteins and edges (lines) denote interactions. The color of each nodes represents the nature of the interactions, e.g., known or predicted interaction. A detailed description of the nodes and the edges can be found at the STRING data base (http://version10.string-db.org/help/getting_started/).

Figure S7

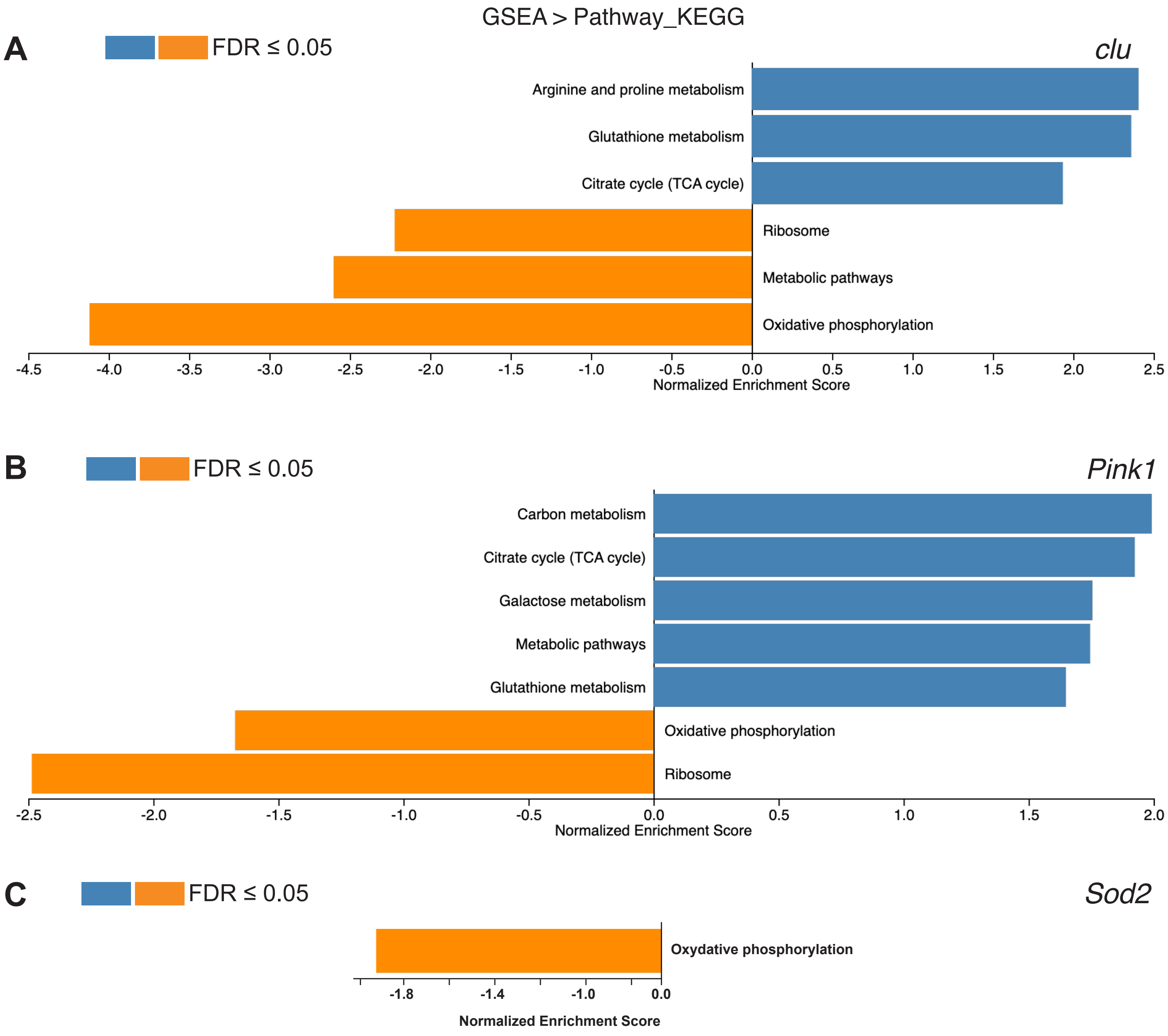

Supplemental Figure S7. The Gene Set Enrichment Analysis (GSEA) for less and more abundant proteins from *clu* (A), *Pink1* (B), and *Sod2* (C) mutants. KEGG Pathway analysis shows enrichment of pathways from less abundant proteins (in orange bars) and more abundant proteins (in blue bars). The names of each pathway are indicated next to each bar. The normalized enrichment scores are shown on the y-axis. Parameters for enrichment analysis are as follow: Minimum number of IDs in the category: 5, Maximum number of IDs in the category: 2000, Significance Level: FDR < 0.05, Number of permutations: 1000. FDR = False Discovery Rate.

Figure S8

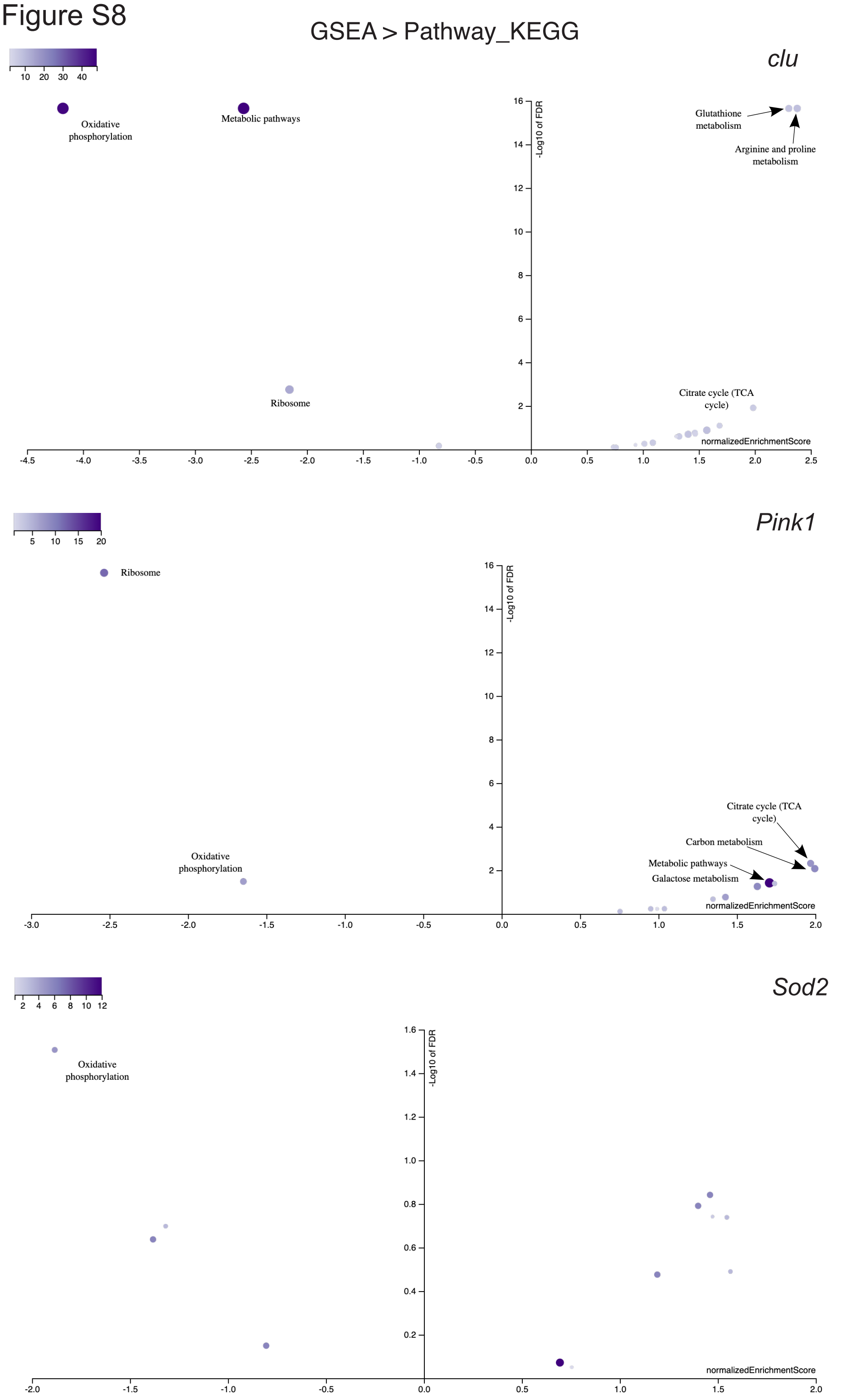

Supplemental Figure S8. The Gene Set Enrichment Analysis (GSEA) for less and more abundant proteins from *clu* (A), *Pink1* (B), and *Sod2* (C) mutants. KEGG Pathway analysis shows enrichment of pathways from less abundant proteins using volcano plots. The names of each pathway are indicated next to each dot. Statistically insignificant pathways are shown as unlabeled dots. The -Log10 of FDR is shown on the x-axis and the normalized enrichment scores are shown on the y-axis. Parameters for enrichment analysis are as follow: Minimum number of IDs in the category: 5, Maximum number of IDs in the category: 2000, Significance Level: FDR < 0.05, Number of permutations: 1000. FDR = False Discovery Rate.

Figure S9

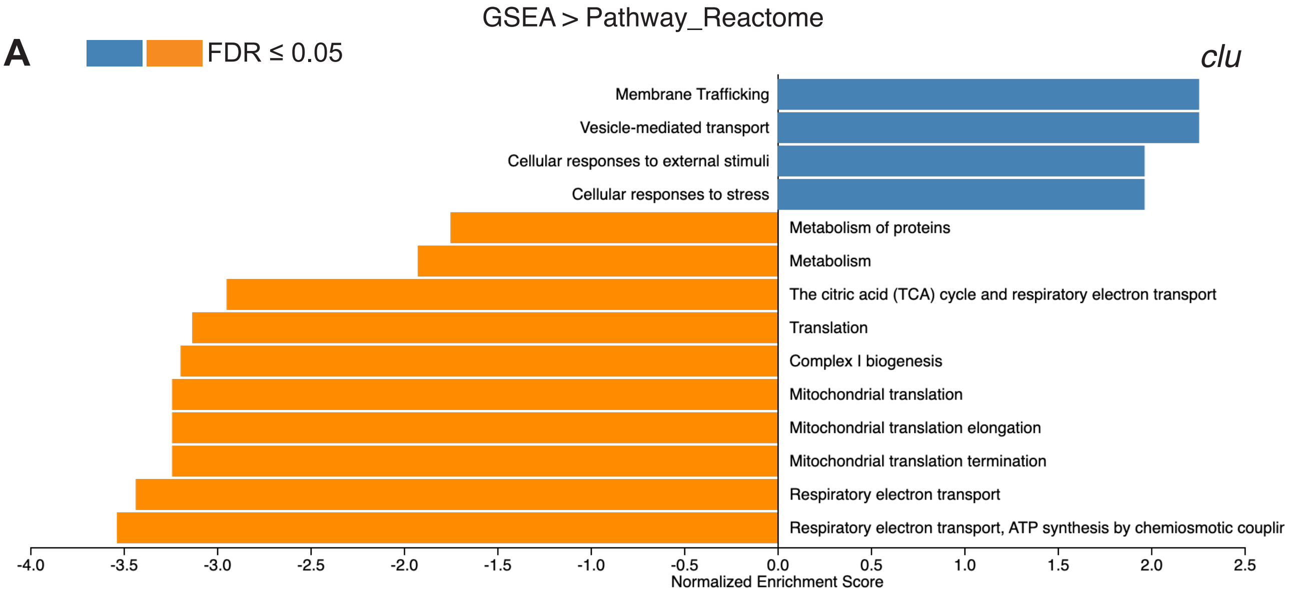

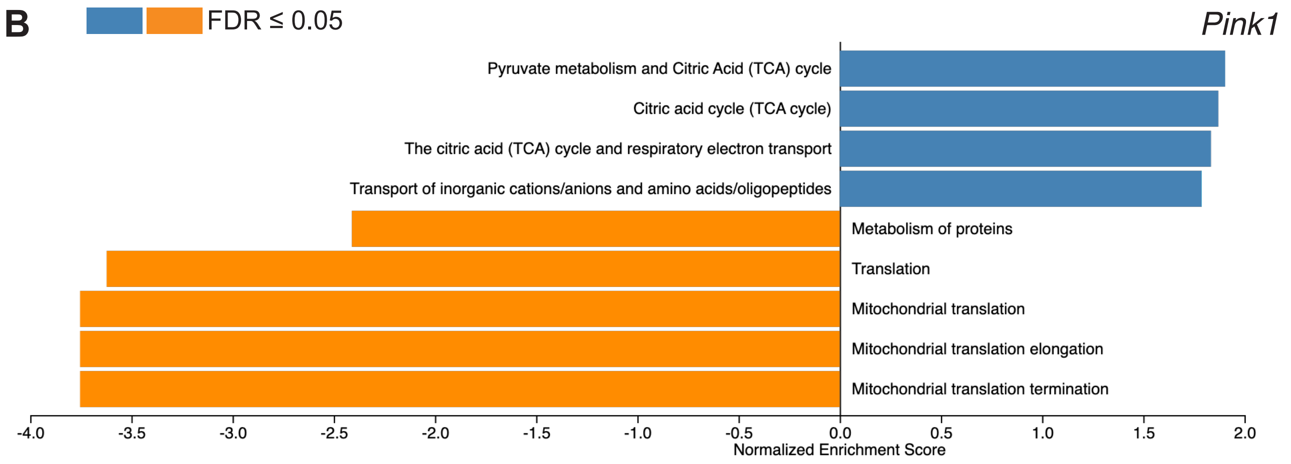

Supplemental Figure S9. The Gene Set Enrichment Analysis (GSEA) for less and more abundant proteins from *clu* (A) and *Pink1* (B). Reactome Pathway analysis shows enrichment of pathways from less abundant proteins (in orange bars) and more abundant proteins (in blue bars). Reactome Pathway analysis did not find any significant changes for Sod2 mutants. The names of each pathway are indicated next to each bar. The normalized enrichment scores are shown on the y-axis. Parameters for enrichment analysis are as follow: Minimum number of IDs in the category: 5, Maximum number of IDs in the category: 2000, Significance Level: FDR < 0.05, Number of permutations: 1000. FDR = False Discovery Rate.

Figure S10: List of primers used one step RT-PCR analysis

**Primers**	**Sequences**
Tom20 forward	CACCATGATTGAAATGAACAAAACTGCAATCGGC
Tom20 reverse	TTCGAGGTCGTCGATACTTGCG
ND-19 forward	CACCATGGTCATCACCAACAACAC
ND-19 reverse	CTCCAGCCAGTGGAAGCGGGAACC
ND-ASHI forward	CACCATGTCGGCGTTTGTGAAAAC
ND-ASHI reverse	CTTCTCGAAGGTGTAGTGC
ND-SGDH forward	CACCATGGTCGGTTGGAGCCGTTTGC
ND-SGDH reverse	GTCTCCGCGCAGAGCCTCCAG
UQCR-14 forward	CACCATGTCGAACTATATTGCCAG
UQCR-14 reverse	GTGGATCTTTTCCCAGTCCTCACG
COX5B forward	CACCATGGCATCGATCTGTGGACGC
COX5B reverse	AACAGCTGCCTTCTCCACCAGC
ATPsynCF6 forward	CACCATGCTGTCGCAATCCCTGCTG
ATPsynCF6 reverse	CTGTGGGGCCTGGGTGATGGGATC
ND-42 forward	CACCATGACCGCCGTGTTCCGCGTAGG
ND-42 reverse	TCGAGGACAACACCCTGGCCGG
ND-23 forward	CACCATGTCGCTAACTATGCGAATTTTCACC
ND-23 reverse	AGCTCCTCGTGCGTCTCCGTGG
COX4 forward	CACCATGGCCCTGCGACTACTCAACAG
COX4 reverse	TGGAGGTCAATCCGGTGACGGGG
Levy forward	CACCATGTCCGCTATTCTAAACCACGC
Levy reverse	GTGCTCGTAGCCGTCGGGCAGGG
UQCR-Q forward	CACCATGCGTCTATCCTCGATCCTG
UQCR-Q reverse	TTCGTCGTTCGCGTAGTCAGCGG
mRpS16 forward	CACCATGTCTCTATCGCCAGCCAGTGG
mRpS16 reverse	TGCGGTTGATTCCGCCTTTTCTGG
HSP22 forward	CACCATGCGTTCCTTACCGATGTTTTGG
HSP22 reverse	CTGACTGGCGGCTTTGTCATTTGGC
